# On the psychometric evaluation of cognitive control tasks: An Investigation with the Dual Mechanisms of Cognitive Control (DMCC) battery

**DOI:** 10.3758/s13428-023-02111-7

**Published:** 2023-04-11

**Authors:** Jean-Paul Snijder, Rongxiang Tang, Julie M. Bugg, Andrew R. A. Conway, Todd S. Braver

**Affiliations:** 1 Department of Psychology, Heidelberg University, Heidelberg, Germany; 2 Department of Psychology, Washington University in St. Louis, St. Louis, MO USA; 3 Division of Behavioral & Organizational Sciences, Claremont Graduate University, Claremont, CA USA

**Keywords:** Cognitive control, Reliability, Individual differences, Dual Mechanisms of Control, Hierarchical Bayesian modeling

## Abstract

The domain of cognitive control has been a major focus of experimental, neuroscience, and individual differences research. Currently, however, no theory of cognitive control successfully unifies both experimental and individual differences findings. Some perspectives deny that there even exists a unified psychometric cognitive control construct to be measured at all. These shortcomings of the current literature may reflect the fact that current cognitive control paradigms are optimized for the detection of within-subject experimental effects rather than individual differences. In the current study, we examine the psychometric properties of the Dual Mechanisms of Cognitive Control (DMCC) task battery, which was designed in accordance with a theoretical framework that postulates common sources of within-subject and individual differences variation. We evaluated both internal consistency and test–retest reliability, and for the latter, utilized both classical test theory measures (i.e., split-half methods, intraclass correlation) and newer hierarchical Bayesian estimation of generative models. Although traditional psychometric measures suggested poor reliability, the hierarchical Bayesian models indicated a different pattern, with good to excellent test–retest reliability in almost all tasks and conditions examined. Moreover, within-task, between-condition correlations were generally increased when using the Bayesian model-derived estimates, and these higher correlations appeared to be directly linked to the higher reliability of the measures. In contrast, between-task correlations remained low regardless of theoretical manipulations or estimation approach. Together, these findings highlight the advantages of Bayesian estimation methods, while also pointing to the important role of reliability in the search for a unified theory of cognitive control.

Cognitive control refers to the set of processes involved in deliberate regulation of information processing to facilitate goal-directed behavior (Miller & Cohen, 2001). Nearly a half-century of research in cognitive psychology has been devoted to the development of experimental task paradigms designed to investigate the processes involved in cognitive control (Posner & Snyder, 1975). Examples from this literature include the Stroop, Simon, flanker, stop-signal, cued task-switching, AX-CPT, and certain variants of the Sternberg item-recognition task. Although these tasks vary along a number of dimensions, one common element to them all is that they involve the utilization of task rules or prior contextual information to resolve response conflict (i.e., competition between task-relevant and automatic response tendencies). How fast and/or accurately the conflict is resolved has been treated as an indicator of cognitive control function. Most of the research in this literature has focused on detailed investigation of individual tasks and “benchmark findings” (e.g., the Stroop interference effect), as a means of testing theories and models regarding core mechanisms of cognitive control (Braem et al., 2019; Bugg, 2012; Kiesel et al., 2010; Verbruggen & Logan, 2009). However, more recent work has focused on the question of whether cognitive control can be considered a domain-general construct, with individuals varying systematically (i.e., in a trait-like fashion) in cognitive control functioning. This shift in the literature has prompted a focus on analyses and measurement of individual differences in cognitive control tasks and batteries (von Bastian et al., 2020).

The current study is situated relative to other recent attempts investigating the measurement of individual differences in cognitive control function (Friedman & Miyake, 2017; Frischkorn et al., 2019; Paap & Sawi, 2016; Rey-Mermet et al., 2018; Whitehead et al., 2019). Specifically, we focus on one of the key issues that has become of recent interest and controversy within this literature; namely, whether it is fundamentally problematic to utilize classic cognitive control tasks, which were developed within the tradition of experimental psychology, to assess individual differences in control functions (Cooper et al., 2017; Hedge, Powell, & Sumner, 2018b; Tucker-Drob, 2011). The cognitive control tasks developed from the experimental tradition are popular because their effects replicate under a wide variety of research settings and task conditions. This success is largely attributable to a combination of low between-subject variance and high within-subject variance. Unfortunately, an individual differences approach thrives under opposite conditions, i.e., high between-subject and low within-subject variance. As a result, when these tasks are used in individual differences research, the measures have often been found to be inconsistent and unreliable, which has been recently termed “the reliability paradox” (Hedge, Powell, & Sumner, 2018b; Kucina et al., 2022; Rey-Mermet et al., 2018; Rouder & Haaf, 2019).

## Dual mechanisms of cognitive control

The development of the Dual Mechanisms of Cognitive Control (DMCC) project and task battery (Braver et al., 2021; Tang et al., 2022) was in part motivated by this paradox. A key distinguishing feature of the DMCC battery is that the tasks included in the battery were specifically designed to test the Dual Mechanisms of Control theoretical framework. This framework postulates distinct proactive and reactive modes of control (Braver, 2012; Braver et al., 2007), that may reflect key dimensions of individual variation in control function. The Dual Mechanisms of Control account provides a theoretical framework that decomposes cognitive control into two qualitatively distinct mechanisms – proactive control and reactive control (Braver, 2012; Braver et al., 2007). Proactive control refers to a sustained and anticipatory mode of control that is goal-directed, allowing individuals to actively and optimally configure processing resources prior to the onset of task demands. Reactive control, by contrast, involves a transient mode of control that is stimulus-driven, and relies upon retrieval of task goals and the rapid mobilization of processing resources following the onset of a cognitively demanding event (Braver, 2012; Braver et al., 2007). In other words, proactive control is preparatory in nature, while reactive control operates in a just-in-time manner. The DMCC task battery includes conditions that are designed to experimentally and independently bias subjects towards the use of proactive and reactive control modes.

In contrast to the selection of tasks used in prior work, we explicitly developed the DMCC task battery to more closely exemplify an integrated experimental/correlational approach first advocated by Cronbach (1957). As Cronbach (1957) articulated, experimental evidence is standardly utilized to inform normative models of the structure and function of cognitive abilities, while correlational/differential data is used to investigate individual differences in those abilities and their role in real-world behavior. Ideally, the experimental and differential approaches inform each other, allowing for a theoretical framework that integrates different kinds of empirical evidence and accounts for inter-individual differences in terms of intra-individual psychological processes.

### Experimental companion paper

We have conducted a systematic validation of the full task battery in terms of its behavioral characteristics. In the current paper, we focus on the psychometric properties of the task battery and its utility for individual difference analyses. Conversely, in a recently published companion paper (Tang et al., 2022), we focused on group effects, testing for dissociations between behavioral markers of proactive and reactive control. The experimental companion paper provides an extensive description of the tasks, manipulations and their rationale, and data-gathering procedures (additional rationale for the tasks is also provided in Braver et al., 2021). Rather than providing a full duplication of this information in the current paper, we report only pertinent methodological details, along with a slightly expanded description in Appendix 3. Interested readers are thus referred to Tang et al. (2022) or the Appendix for this information.

A key element of Tang et al. (2022) was to provide a comprehensive introduction to the DMCC battery and the associated dataset acquired with it, highlighting both its convergent (cross-task) and divergent (discriminant) validity. Tang et al. (2022) reported analyses demonstrating that dependent measures show both consistent proactive and reactive effects across tasks within the battery, with 20 out of 26 of the key theoretical predictions being confirmed. Specifically, in terms of convergent validity, the experimental manipulations were generally effective in producing group-level shifts in proactive control and reactive control in each task, suggesting consistent across-task sensitivity to changes in cognitive control demands due to the experimental manipulations. In terms of divergent validity, there were clear patterns of double dissociation, in that the behavioral markers of proactive and reactive control could effectively be distinguished in all tasks.

For the current paper, we utilize the DMCC battery as a vehicle from which to evaluate whether the cognitive control tasks included in the battery can measure individual differences reliably. As the DMCC battery utilizes theoretically motivated task manipulations, a critical question is whether such manipulations impact their sensitivity to individual variation in task performance. According to classical test theory, the proportion of variability that is specifically related to the construct of interest (in this case, cognitive control demand) is referred to as “true score variance” (Novick, 1966). Tasks that have high true-score variance are also expected to exhibit stronger reliability and validity (Chapman & Chapman, 1978). Interestingly, in prior work focusing on only one task in the DMCC battery, the AX-CPT, we demonstrated differential sensitivity to individual differences in working memory capacity in the proactive control mode, relative to baseline and reactive modes (Gonthier et al., 2016; Lin et al., 2022). This finding is consistent with the hypothesis that, by isolating proactive and reactive control modes within the DMCC battery, we have increased true-score variance in the task metrics of interest. Concretely, using Bayesian linear mixed effect models we found that AX-CPT measures theoretically linked to proactive control (A-cue bias, BX RT interference, d’prime-context) were selectively stronger in the proactive condition (i.e., the condition experimentally encouraging proactive control), even when statistically controlling for variance in the baseline and reactive conditions.

Nevertheless, it is generally accepted that the most rigorous approach to assess sensitivity to individual differences is through a comprehensive analysis of psychometric reliability. Consequently, our goal for the current paper is to provide such a comprehensive analysis for the DMCC task battery. Nevertheless, this type of analysis can be particularly complex within the domain of experimental tasks assessing cognitive control. To illustrate this complexity more fully, in the sections that follow, we briefly review the literature on individual differences in cognitive control, the approaches used to assess such individual differences, and the measurement challenges associated with the evaluation of task reliability in this domain.

## Measuring individual differences in cognitive control

Individual differences in cognitive control are associated with several important real-world outcomes, including psychopathology (Snyder et al., 2015), impulsivity (Sharma et al., 2014), addiction (Hester & Garavan, 2004), and age-related cognitive decline (Hasher et al., 1991). The ability to engage cognitive control is strongly linked to working memory capacity, which is associated with a broad range of outcomes, including academic achievement (Alloway & Alloway, 2010; Gathercole et al., 2003), reading comprehension (Daneman & Carpenter, 1980), mathematical ability (Ramirez et al., 2013), and multi-tasking (Redick et al., 2016). Cognitive control plays an important role in contemporary theories of intelligence. By some accounts, cognitive control is considered to be the primary source of variance in overall cognitive ability (Engle & Kane, 2004; Kovacs & Conway, 2016).

Despite these established findings, a major concern in the field is that the tasks used to measure cognitive control often show poor reliability and weak correlational results. Recently, several research groups reported low task reliabilities and/or weak between-task correlations, especially with respect to tasks thought to index aspects of inhibitory control (Hedge, Powell, & Sumner, 2018b; Rey-Mermet et al., 2018 and Stahl et al., 2014). For example, in the Hedge, Powell, and Sumner (2018b) study, the median *test–retest* reliability across seven classic experimental effects (e.g., Stroop, flanker) was surprisingly low, with a median of .40. Similarly, across multiple studies, the correlation between flanker (Eriksen & Eriksen, 1974) and Stroop (Stroop, 1935) effects was below .20 (Draheim et al., 2020; Gärtner & Strobel, 2019; Hedge, Powell, & Sumner, 2018b; Rey-Mermet et al., 2018). Based on these and other similar dismal correlational results, Rey-Mermet et al. (2018) concluded, “we should perhaps stop thinking about inhibition as a general cognitive construct”.

A fundamental question raised by these findings is whether classic experimental tasks are suitable for examining individual differences (Tucker-Drob, 2011). As mentioned, experimental tasks are designed to maximize variance across conditions (within-subject variance) and minimize between-subject variance. This is clearly problematic for researchers interested in studying individual differences. Also, measures of cognitive control that are obtained from experimental tasks (e.g., Stroop effect) are often based on difference scores (e.g., Incongruent RT – Congruent RT). This poses a further challenge, because the reliability of difference scores is constrained by the reliability of the two condition scores and is attenuated by the correlation between the two condition scores. As a result, difference score measures of cognitive control often suffer from low reliability (Cronbach & Furby, 1970; Hedge, Powell, & Sumner, 2018b). Finally, the correlation between any two measures of cognitive control (e.g., Stroop effect and flanker effect) will be constrained by the amount of between-subject variance and the reliability of each measure, so conclusions drawn from correlational studies using experimental tasks may also be inconsistent and unreliable (Nunnally Jr., 1970; Parsons et al., 2019; Spearman, 1904). Thus, based on these reliability issues, it could be argued that the examination of relationships between individual difference measures extracted from experimental tasks (i.e., between-task relationships) maybe highly problematic in a foundational way (Spearman, 1910).

## The measurement and reporting of reliability

### Definitional confusion

In addition to the concerns regarding the measurement of individual differences in experimental tasks, there are numerous issues related to the measurement and reporting of reliability itself. One of the most important issues is that reliability is actually only infrequently reported in cognitive experimental research (Parsons et al., 2019). As described above, part of the reason may be that experimental researchers often have less fluency and familiarity with psychometric issues, including a confusion regarding the technical meaning of reliability as it is utilized in psychometrics. A potential source of confusion may be that the term “reliable” has different meanings in experimental versus correlational psychology. An experimental manipulation is “reliable” when the intended effect is replicated across multiple studies (in different labs, with different stimuli, etc.). In contrast, an individual differences measure is considered “reliable” when it consistently gives similar rankings for individuals. This lack of concern regarding psychometric reliability may be one of the reasons it has not been typically considered as a source of poor correlational results (Flake et al., 2017; Hussey & Hughes, 2020). Conversely, based on this confusion, some results may have been erroneously reported as replicable and generalizable, perhaps propagating false standards in the field (e.g., the replication crisis).

### Problems with reporting reliability: Internal consistency

A second and more fundamental issue is that there is currently no gold-standard procedure for estimating reliability, particularly for experimental tasks (Parsons et al., 2019). Consequently, even when reliability is reported for these tasks, it is not always clearly communicated what estimation approach was utilized, which can lead to erroneous assumptions regarding the reliability of a particular experimental measure. Relatedly, although many statistical software packages supply functionality for computing reliability, these packages assume that the data conforms to analysis-specific assumptions which may not be valid for common experimental tasks and measures. An illuminating example can be seen in the case of Cronbach’s alpha, a measure of internal consistency, which is probably the most common and well-known index of reliability. Alpha is commonly derived by averaging the correlations between each item (trial) and the sum of the remaining items (trials). The default method offered in statistical software packages calculates alpha based on the assumption that items and the order of the items are identical for all subjects. Furthermore, it is assumed that each item measures the same underlying construct, to varying degrees, as a function of item difficulty and discriminability. In survey research, this is often the case. However, in cognitive-behavioral tasks, trial order is often random. More concerning, the cognitive processes involved in task performance may change across trials, as a function of practice, fatigue, sequential effects, or strategy development/deployment. If these issues are ignored, which is typically the case, then reliability estimates may not be accurate or valid. Hence, Cronbach’s alpha is unsuitable for tasks designed to measure individual differences in cognitive control.

There are other issues with the use of Cronbach’s alpha as a measure of split-half reliability. Formally, if the assumptions above hold, Cronbach’s alpha is identical to the average of all correlations between two halves of the data. However, split-half reliability is most commonly calculated in a sample by splitting the data – once – into the first and second half or even- and odd-numbered trials, and computing the correlation between these measures. However, it has been demonstrated that split-half reliabilities based on these kinds of simple split methods are unstable. Enock et al. (2012) showed that reliabilities vary depending on which trials were used in the partitioning. They recommend applying multiple random splits to the data to generate multiple split-half reliability estimates and then taking the average of all split-half estimates as the overall reliability estimate (Enock et al., 2012; Parsons et al., 2019). This permutation-based method for calculating split-half reliability approximates Cronbach’s alpha (Cronbach, 1951), while simultaneously avoiding the pitfalls described above. However, another important issue is that splitting the number of observations in half leads to underestimation. The Spearman–Brown (prophecy) formula can be applied to correct for this underestimation (corrected reliability = [2*reliability] / [1+reliability]), yet this correction approach is not well known or frequently utilized.

### Problems with reporting reliability: Test–retest

A third important issue is that internal consistency reliability is not the same as test–retest reliability. The measurement and utilization of test–retest reliability can be used when the same individuals are measured on the same test on two or more assessment occasions. Test–retest reliability indices estimate the degree to which the measure provides stable rankings of individuals across time. The most well-established index of test–retest reliability is the intraclass correlation coefficient (ICC), which indicates how well the measurements consistently rank-order the subjects. However, one of the complexities of ICC, which has also created some confusion in its usage, is that there are ten distinct forms available (Mcgraw & Wong, 1996). Yet only two forms are particularly pertinent for measures from cognitive experimental tasks (for a more in-depth discussion see Koo and Li (2016).

A critical distinction in the use of ICC estimates is whether reliability is based on either consistency or the absolute agreement between the two measurements (e.g., the relationship). A consistency relationship is not affected by systematic changes (e.g., practice effects, learning between measurements) and only the consistency of the rank-order is rated. An absolute agreement relationship is one in which the two measurements are expected to be identical in rank-order *and* in value (e.g., session mean), in other words, this relationship is affected by systematic differences. For example: these two measurements {1,2,3}, {4,5,6} would have a perfect consistent relationship (ICC (3,1) = 1.00), but the measurements would be far from absolute agreement (ICC (2,1) = .09). Thus, the type of relationship expected is a critical consideration when deciding which form of ICC to use when calculating test–retest reliability of samples from cognitive behavioral measures. If the researcher expects systematic differences between measurement occasions (e.g., practice effects), then the preferred form of ICC is the type termed ICC (3,1) in the standard terminological conventions developed by Shrout and Fleiss (1979). Conversely, if systematic differences between occasions should be considered to be problematic for the reliability of a measure, then the ICC (2,1) type should be selected. Importantly, it is necessary for the researcher to explicitly specify which type of ICC was used for calculation, and the rationale for selection, so that no ambiguity exists with regard to interpretation.

### Traditional versus hierarchical Bayesian approaches

A final issue is that traditional analytic approaches, such as ICC, may be sub-optimal, and actually even inappropriate, when calculating test–retest reliability in cognitive experimental tasks. Specifically, traditional approaches to test–retest reliability treat summary score measures (sometimes referred to as mean point-estimates; MPE) as representative indicators of performance; yet these measures do not consider trial-to-trial variability, which in itself could be an important source of individual differences (Haines et al., 2020; Lee & Webb, 2005; Rouder & Haaf, 2019; Rouder & Lu, 2005). Indeed, Rouder and Haaf (2019) have presented evidence that by ignoring trial-to-trial variability, test–retest reliability is “greatly” attenuated (see also von Bastian et al., 2020). As an alternative approach, newer analytic methods, involving hierarchical modeling (also termed multilevel or linear mixed effects modeling), have been introduced for measuring reliability, which simultaneously assess between- and within-subject (i.e., trial-to-trial) variation. Hierarchical modeling is a statistical framework for modeling data that have a natural hierarchical structure. For example, data from cognitive-behavioral tasks often have trials within subjects and subjects within groups. By restructuring a model hierarchically, all individuals are considered in two contexts: in isolation, to determine how behavior varies across trials, and as a contributing member of a group, to determine how behavior varies across the group. This increases the number of available parameters from one (i.e., MPE) to multiple (e.g., mean, standard deviation). The model can now distribute uncertainty (e.g., measurement error) that exists in the data over those multiple parameters, which results in more precise estimates at both the individual and group levels (Kupitz, 2020). In particular, hierarchical models provide the means to appropriately correct for the attenuation of reliability that may occur when using more traditional methods.

Additionally, these recent efforts have also pointed to the advantages of hierarchical *Bayesian* models (HBM), relative to classic “frequentist” approaches. A key advantage of the HBM approach is that it can be used to specify a single model that *jointly* captures the uncertainty at both the individual- and group-level. Even in a typical study that involves a modest number of subjects, each performing a limited number of trials with the observed data confounded by measurement error, HBM can provide reasonable estimates of performance, by assuming that the data are generated from a population of infinite trials (Raudenbush & Bryk, 2002; Snijders & Bosker, 1999). A second advantage of HBM is it enables explicit specification of distributions and associated parameters, which best fits a generative approach in which individual trial performance measures are thought to reflect samples drawn from these distributions. Among others, Haines et al. (2020) highlight the advantages of generative models, by suggesting that models more accurately “simulate data consistent with true behavioral observations *at the level of individual participants*”. In contrast to HBM, frequentist methods of accounting for hierarchical sources of variability, such as structural equation modeling or classical attenuation corrections, do not provide a natural framework for generative modeling (Kurdi et al., 2019; Westfall & Yarkoni, 2016).

## Introduction summary

This brief review of the current state of research on individual differences in cognitive control function suggests that a barrier to progress is the lack of knowledge on the part of researchers coming from the cognitive experimental tradition, regarding some of the psychometric complexities associated with individual difference measurement. A potential remedy is for researchers to be more explicit regarding assumptions that are being utilized regarding measurement method. Part of this explicitness relates to the reporting of measurement reliability and the analytic approach used for estimation. Moreover, when possible, estimates of both internal consistency (i.e., permutation-based split-half) and temporal stability (i.e., test–retest, ICC) forms of reliability should be assessed and reported. Finally, further investigation and comparison is needed between traditional frequentist and Bayesian approaches to estimation, since the use of Bayesian approaches in individual differences analyses is a relatively new development in the literature.

## Current study

The overarching goal of the current study is to test whether a task battery designed in accordance with a unifying theoretical framework, can more successfully bridge the divide between experimental and differential approaches in cognitive control research. Here we provide an evaluation focused on the utility of cognitive control measures for individual differences research purposes; specifically, we examine the psychometric issues described above within the context of the DMCC battery. In particular, a key objective associated with the development of the DMCC battery was to examine how experimental manipulation of cognitive control mode affects individual difference properties of classic cognitive control tasks (Stroop, AX-CPT, cued task-switching, and Sternberg). A key question of interest was whether these task manipulations would allow for more reliable measurement of individual differences in cognitive control function (Cooper et al., 2017). More specifically, by employing task variants that selectively isolate proactive and reactive control modes, respectively, the reliability of mode-specific individual variation can be estimated. Moreover, it is possible that mode-specific individual variation is associated with reduced measurement error. Tang et al. (2022) provide initial support for this hypothesis by demonstrating the convergent and divergent validity of the proactive and reactive control indices, in terms of the robustness of group-average experimental effects. Here we test whether the tasks also demonstrate strong psychometric reliability as individual difference measures of cognitive control ability. Consequently, we sought to assess task reliability in a systematic and comprehensive manner.

Another important focus of the paper was to compare traditional and the newer HBM approaches described above, for the assessment of psychometric reliability. The first set of analyses thus report reliability, both internal consistency and test–retest, employing traditional approaches based on summary score measures (MPEs) from each subject. In contrast, for the second set of analyses we implement hierarchical methods to incorporate modeling of trial-to-trial variability (i.e., individual-level standard deviation) (Rouder & Haaf, 2019). Specifically, we directly compare the traditionally derived test–retest reliability measures with those derived from the HBM approach. Our second hypothesis was that traditional approaches would substantially under-estimate the degree of reliability present in cognitive control tasks, replicating prior findings (Rouder & Haaf, 2019).

Our third hypothesis was that Bayesian parameter estimates, if more reliable, would also be more suitable for individual differences analyses that address the question of whether cognitive control can be considered a domain-general construct (i.e., with individuals varying in a consistent, trait-like manner). Consequently, as a final analysis, we examined correlations present in the DMCC task battery, both within task (i.e., the relations between the baseline, proactive, and reactive variants of each task), and across each task with the same control mode condition (i.e., the relations between the different task paradigms).

## Method

### Subjects

Subjects were recruited via the Amazon Mechanical Turk (MTurk) online platform. Our inclusion criteria required subjects to be physically in the United States of America, have an Amazon HIT approval rate of or greater than 90%, and had prior to our study completed at least 100 online experiments offered on MTurk. Subjects were excluded if they had participated in any of our other experiments with DMCC tasks, or if they were Mac OS users (due to limitations in the testing software, particularly for recording vocal reaction times in the Stroop task). After reading a description of the study that indicated its multi-session nature and time commitment, 225 interested subjects accessed a link which allowed them to review and sign the consent form. Only 128 subjects completed the entire study.

After consent was given, the web-links for the first session of the study were made available on MTurk. Subjects were not restricted with regard to age range^1^, and as such a wide range was included in the sample (*N* = 128; 22–64, M = 37.11, SD = 9.90; 82 females, 46 males).

### Design and procedure

The study protocol consisted of 30 separate testing sessions that subjects completed in a sequential manner (15 for the test phase, and another 15 for retest). Subjects completed the sessions at a rate of five per week, i.e., taking 6 weeks to complete the full protocol. Baseline task variants were completed during the first and fourth week, the reactive task variants during the second and fifth week, and the proactive task variants were completed during the third and sixth week. Each session lasted approximately 20–40 min in duration, with the exception of the first session, which was 1 h in duration (and included a Stroop practice to validate operation of vocal response recording, along with a battery of demographic and self-report questionnaires). To both incentivize and prorate study completion, completion of the first session of both test and retest phases resulted in a $4 payment, each subsequent session was paid $2, with the exception of session 6 and 11, which were paid $4 for each. Additional bonuses of $20 were paid for completion of the test phase and $30 for full study completion. Together, successful completion of the entire protocol resulted in a payment of $122.

For each completed session, the experimenter checked for overall accuracy and completion of each task and questionnaire to make sure that subjects were complying with instructions and maintaining sufficient attention to the task. A criterion of 60% accuracy and response rate was used to determine whether the data would be included, and the subject invited to remain in the study. For each task that did not meet the criterion, the experimenter attempted to communicate with the subject first to determine if they had trouble understanding the instructions or had technical difficulties. If so, the subject was given a second chance to complete the task before a designated deadline. Within each of the test and retest phases, sessions were conducted in a fixed order for all subjects.

### Task paradigms

**Fig. 1:**
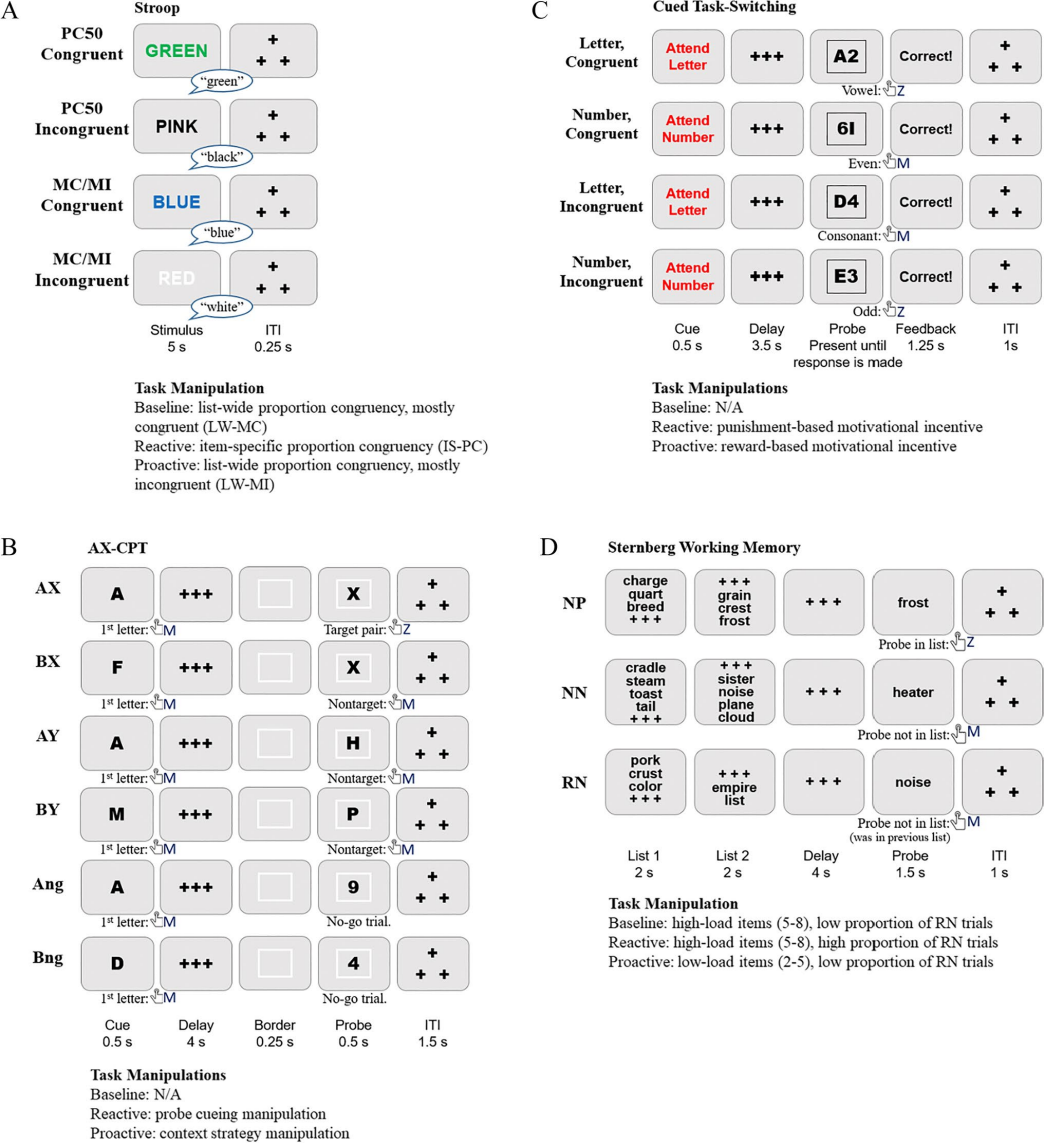
DMCC task paradigms and overview of session manipulations. *Note. PC* = proportion congruency; *MC/MI* = mostly congruent/mostly incongruent; *ITI* = intertrial interval; *Ang* = no-go trials with an A cue; *Bng* = no-go trials with an B cue; *NP* = novel positive; *NN* = novel negative; *RN* = recent negative. For a more detailed description, see Appendix 3 and Tang et al., 2022

Here we present a schematic representation of the tasks and their manipulations (see Fig. 1). The full task descriptions are provided in Tang et al. (2022). Additionally, tasks, experimental design, and manipulation rationale are also provided in Appendix 3 within the current manuscript.

### Data pre-processing

To facilitate comparison of results across task paradigms, subjects who failed to complete all 30 sessions were not included in the analyses reported here; data from 128 subjects entered the pre-processing stage. For all tasks: correct trials with reaction time (RT) values faster than 200 ms and slower than three standard deviations above the mean RT at the trial type level (i.e., trial type within session, phase, and subject) were removed. This resulted in the removal of 1.7% of Stroop RT trials, 2.9% AX-CPT RT trials, 1.5% of task-switching RT trials, and 0.8% of Sternberg RT Trials.

After removal of RT trials, each condition at the trial type level was inspected to ensure that no more than half of completed correct trials were removed during the pre-processing. Subjects passing the criterium were deemed to have enough trials to enter further analyses, but this criterium was ultimately arbitrary. Any subject that had a condition removed based on the criterium was removed from the task-specific analyses (i.e., test–retest), but not from the experiment. Hence, the disparity in sample sizes reported in these analyses. This step resulted in the removal of ten subjects from the AX-CPT data, 24 from the Sternberg data, and 0 from the Stroop or task-switching data.

For error rates, following Gonthier et al. (2016), we set a 40% error rate cutoff for the AX-CPT task. Common error rate cutoffs for the remaining tasks were less well documented and we utilized multiverse analyses of different cutoff values (i.e., error rate of 20, 30, 40, and 50%) to determine fitting thresholds. Examining and visualizing the remaining data at each cutoff for the remaining tasks, revealed that a 40% cutoff removed severe outliers, while maximizing subject retention. Applying a 40% cutoff at the trial type level across all task resulted in: one subject removed from the Stroop data, no subjects removed from the AX-CPT data, two subjects removed from the task-switching data, and 24 subjects removed from the Sternberg data. The high number of subjects removed from both the RT and error rate Sternberg data is due to the low number of critical novel negative trials in the reactive session, see further comments in the discussion section. Finally, for the correlational analyses in the current study (i.e., Figs. 3 and 4, Tables 4 and 5), complete data per bivariate analysis were used. Additionally, data entering the correlations were tested for bivariate outliers using Mahalanobis distance with a cutoff of 10.828 (alpha = .001, and *df* = 1).

### Data analyses

We assessed psychometric reliability (both split-half and test–retest) of the measures taken from the four DMCC tasks within each of three sessions (e.g., baseline, proactive, reactive). The analyses reported in the main text focused on the critical conditions of the tasks (i.e., Stroop biased condition, task-switching biased condition, Sternberg list-length 5 condition), as defined in Tang et al. (2022). The critical conditions were designed specifically to allow for comparison across tasks and analytic methods. Full descriptive statistics and experimental results by session, task, and trial type for all conditions are reported in Tang et al. (2022). Additional reliability analyses (using traditional approaches only) of other non-critical conditions are reported in the Appendix. In addition to examining the reliability of each critical condition measure, we also examined the strength of correlation between measures, focusing on both within-task, between-condition correlations (e.g., Stroop baseline vs. Stroop proactive) and between-task, same-condition correlations (e.g., AX-CPT reactive vs. Sternberg reactive). If reliability indeed serves as a bottleneck that attenuates the magnitude of between-measure correlations, then improving reliability should dis-attenuate true underlying correlations (given that a relationship exists between the measures).

#### Reliability estimates: Traditional approach

Both internal consistency and test–retest forms of reliability were calculated, based on traditional psychometric approaches. Internal consistency estimates were calculated as permutation-based split-half correlations. The data were repeatedly (5000 permutations) and randomly split into halves, which were then correlated and a Spearman–Brown correction was applied. The estimates reported here are an average of those 5000 corrected correlations. Test–retest reliabilities are reported as intraclass correlation coefficients (ICC). Because practice effects are expected to occur from session to session and from test to retest phases, the ICC relationship parameter was examined as both absolute agreement (ICC(2,1)) and consistency (ICC(3,1)), as per the Shrout and Fleiss (1979) convention. The former is sensitive to changes in the mean between repeated measures, whereas the latter appropriately corrects for such changes. Here, we report both forms for comparison purposes.

#### Reliability estimates: Hierarchical Bayesian model

In addition to the traditional psychometric approach to test–retest reliability estimation, HBM was also used to generatively model the reaction time difference score effects from the four tasks in the Dual Mechanisms of Cognitive Control (DMC) task battery. Specifically, we focused on the Stroop effect, the BX interference effect from the AX-CPT, task-rule congruency effect (TRCE) from the cued task switching task, and the *recency effect* from the Sternberg task. Although the HBM approach works for accuracy measures as well, given concerns regarding difference scores in psychometric analyses (which are traditionally reaction time based), we focused on these measures to determine the potential advantages of the HBM approach. Additionally, these measures are ones that are commonly computed for each task (for more information and rationale on task measures see Appendix 3). Finally, specifying a generative model encapsulates the key assumptions that are shared among the tasks: (1) reaction time cannot be negative; (2) reaction time responses vary around some central tendency (this is ignored with MPE); (3) the central tendency varies per subject; (4) within-individual (i.e., trial-by-trial) variability varies per subject; and (5) reaction time distributions from cognitive-behavioral tasks tend to be right-skewed (Wagenmakers & Brown, 2007).

In the HBM approach, it is important that estimation of test–retest reliability considers trial variability at the individual-level; hence, the individual-level distribution is defined first, followed by the group-level distribution. Given the additional complexity and lower reader familiarity with the HBM approach, we elaborate on how these distributions and parameters are estimated. Individual-level reaction time response distributions are here conceptualized as coming from a lognormal distribution, satisfying the skewed distribution assumption (assumption 5). The distribution is further shaped by mean and standard deviation parameters, which *both* vary per subject and between each condition (satisfying assumptions 2, 3, and 4). Theoretically, the distribution parameters are not expected to vary much between the test and retest phase. However, for test–retest reliability purposes, the model assumes unique distributions for each phase as well.

4 $${\textrm{RT}}_{i,c,p}\sim \textrm{Lognormal}\left({\mu}_{i,c,p},\exp \left({\sigma}_{i,c,p}\right)\right)$$

Formally, in Eq. (1), RT_*i*, *c*, *p*_ is the observed reaction time data for subject *i* = {1, … , *N*}, in condition *c* = {control, interference}^2^, during phase *p* = {test, retest}.

~Lognormal(*μ*_*i*, *c*, *p*_, exp(*σ*_*i*, *c*, *p*_)) signifies that the data are drawn from a generative process producing a skewed distribution (i.e., a lognormal distribution), shaped by a mean and standard deviation parameter for each subject, condition, and phase combination. A lognormal distribution has an asymmetrical spread; more variability is found on the right-side (i.e., slow reaction times) of the central tendency than the left-side (i.e., fast reaction time). Importantly, the lognormal distribution has a property that determines how the mean and standard deviation interact, allowing the model to fit the many different shapes of reaction time distributions produced by the ~ 120 subjects. Wagenmakers and Brown (2007) show that this property adheres to a *law of [reaction] time*, which states that in reaction time performance, the standard deviation increases linearly with the mean. In other words, the slower a subject’s mean reaction time, the more individual-level variability they show. Additionally, to ensure that the individual-level standard deviation parameters are greater than 0, they are exponentially transformed.

Individual-level parameters are informed by group-level parameters, and vice versa. The hierarchy of the model is constructed so that the individual-level distribution parameters from Eq. (1), denoted by *μ*_*i*, *c*, *p*_ and *σ*_*i*, *c*, *p*_, are drawn from group-level multivariate normal distributions (i.e., prior models), with unobserved (i.e., unknown) means and standard deviations (*σ*):

2 $${\displaystyle \begin{array}{cc}\left[\begin{array}{c}\begin{array}{c}{\mu}_{i,c=1,p=1}\\ {}{\mu}_{i,c=1,p=2}\end{array}\\ {}{\mu}_{i,c=2,p=1}\\ {}{\mu}_{i,c=2,p=2}\end{array}\right]& \sim \textrm{MVNormal}\left(\left[\begin{array}{c}\begin{array}{c}{\mu}_{mean,c=1,p=1}\\ {}{\mu}_{mean,c=1,p=2}\end{array}\\ {}{\mu}_{mean,c=2,p=1}\\ {}{\mu}_{mean,c=2,p=2}\end{array}\right],{\textbf{S}}_{\mu}\right)\\ {}\left[\begin{array}{c}\begin{array}{c}{\sigma}_{i,c=1,p=1}\\ {}{\sigma}_{i,c=1,p=2}\end{array}\\ {}{\sigma}_{i,c=2,p=1}\\ {}{\sigma}_{i,c=2,p=2}\end{array}\right]& \sim \textrm{MVNormal}\left(\left[\begin{array}{c}\begin{array}{c}{\sigma}_{mean,c=1,p=1}\\ {}{\sigma}_{mean,c=1,p=2}\end{array}\\ {}{\sigma}_{mean,c=2,p=1}\\ {}{\sigma}_{mean,c=2,p=2}\end{array}\right],{\textbf{S}}_{\sigma}\right)\end{array}}$$

By defining these prior models, the group-level multivariate distribution allows for the pooling of subject-level performance across the four condition and phase combinations. Each of the individual-level parameters, *μ*_*i*, *c*, *p*_ and *σ*_*i*, *c*, *p*_, inform the group-level means and standard deviations, *μ*_mean, *c*, *p*_, *μ*_sd, *c*, *p*_ and *σ*_mean, *c*, *p*_, *σ*_sd, *c*, *p*_, which in turn inform all other individual-level parameters. This mutual interaction creates *hierarchical pooling*, regressing the individual-level parameters towards a group mean (also called *shrinkage* or *regularization*), and increases the precision of Bayesian estimation (Gelman et al., 2013). Bayesian modeling allows for such a “joint model” specification, in which the individual-level and group-level parameters are estimated simultaneously. This embodies the generative perspective (Haines et al., 2020).

Keen observers will notice that the group-level distributions are both modeled as normal, whereas the individual-level distributions are lognormal. Recall that the individual-level standard deviation parameter [Eq. (1); exp(*σ*_*i*, *c*, *p*_)] was exponentially transformed to force it to assume positive values only. Mathematically, when *y* has a normal distribution then the exponential function of *y* has a lognormal distribution. It follows then, that the group-level distribution modeled on the individual-level standard deviation parameter ( exp(*σ*_*i*, *c*, *p*_)) corresponds to a lognormal distribution.

Another key aspect of HBM is the definition of prior probability distribution, which expresses a prior belief about an underlying distribution of interest. Here, parameter estimation is rather robust to prior models, because the priors are rather diffuse and the sample sizes of observed data are relatively large. The prior model for the group-level mean parameters were specified as normal.

3 $${\displaystyle \begin{array}{c}{\mu}_{mean,c,p}\sim \textrm{Normal}\left(0,1\right)\\ {}{\sigma}_{mean,c,p}\sim \textrm{Normal}\left(0,1\right)\end{array}}$$

The prior model for the group-level standard deviations parameters were specified as half-normal (i.e., if *y* is a normal distribution, then | *y* | is a half-normal distribution, folded along the mean with the purpose of consisting of only positive values). Because the individual-level standard deviation parameter is exponentially transformed, the group-level distribution assumes only positive values.

4 $${\displaystyle \begin{array}{c}{\mu}_{sd,c,p}\sim \textrm{Half}-\textrm{Normal}\left(0,1\right)\\ {}{\sigma}_{sd,c,p}\sim \textrm{Half}-\textrm{Normal}\left(0,1\right)\end{array}}$$

To estimate the test–retest reliability, a difference score parameter delta (i.e., ∆) was specified in Stan’s generated quantities code section. To again take the Stroop task as an example, the Stroop effect is incongruent (interference) minus congruent (control) performance. A delta was estimated for the test and retest phase.

5 $${\displaystyle \begin{array}{c}{\Delta }_{i, test}={\mu}_{i, interference, test}-{\mu}_{i, control, test}\\ {}{\Delta }_{i, retest}={\mu}_{i, interference, retest}-{\mu}_{i, control, retest}\end{array}}$$

Then, using the MCMC samples, we correlated delta at test (∆_*i*, *test*_) with delta at retest (∆_*i*, *retest*_), resulting in a posterior distribution of test–retest reliabilities. Test–retest reliability estimates for the delta parameter were calculated for each task and session combination and shown in Fig. 3, indicated as HBM. Importantly, test–retest reliability is calculated as a Pearson *r* correlation between the test and retest phase estimates *r*(∆_1_, ∆_2_). Here, Pearson *r* is chosen over an intraclass correlation coefficient (ICC). In the traditional ICC approach, the within-subject variance (i.e., our model’s *σ*_i_) is still in the mean point-estimates from which the different types of variances needed are calculated. However, ultimately we are interested in the correlation between the delta parameters, which are composed of mu parameters with much of their variance modeled out by the sigma parameter. Hence, a simple correlation suffices and fits our model. This also replicates the generative modeling approach of prior work (i.e., Haines et al., 2020; Rouder & Haaf, 2019).

All model parameters were estimated with Stan (Stan Development Team, 2020b) through an interface in R, called RStan (Stan Development Team, 2020a). All models were fit with three chains of 3000 iterations after 1000 warm-up iterations. For each of the four tasks in the task battery, the model was fit three times (e.g., once for each task-variant), resulting in 12 model fits. From the model fits we extracted three families of parameters: mu, sigma and, most importantly the delta parameters.

**Fig. 2:**
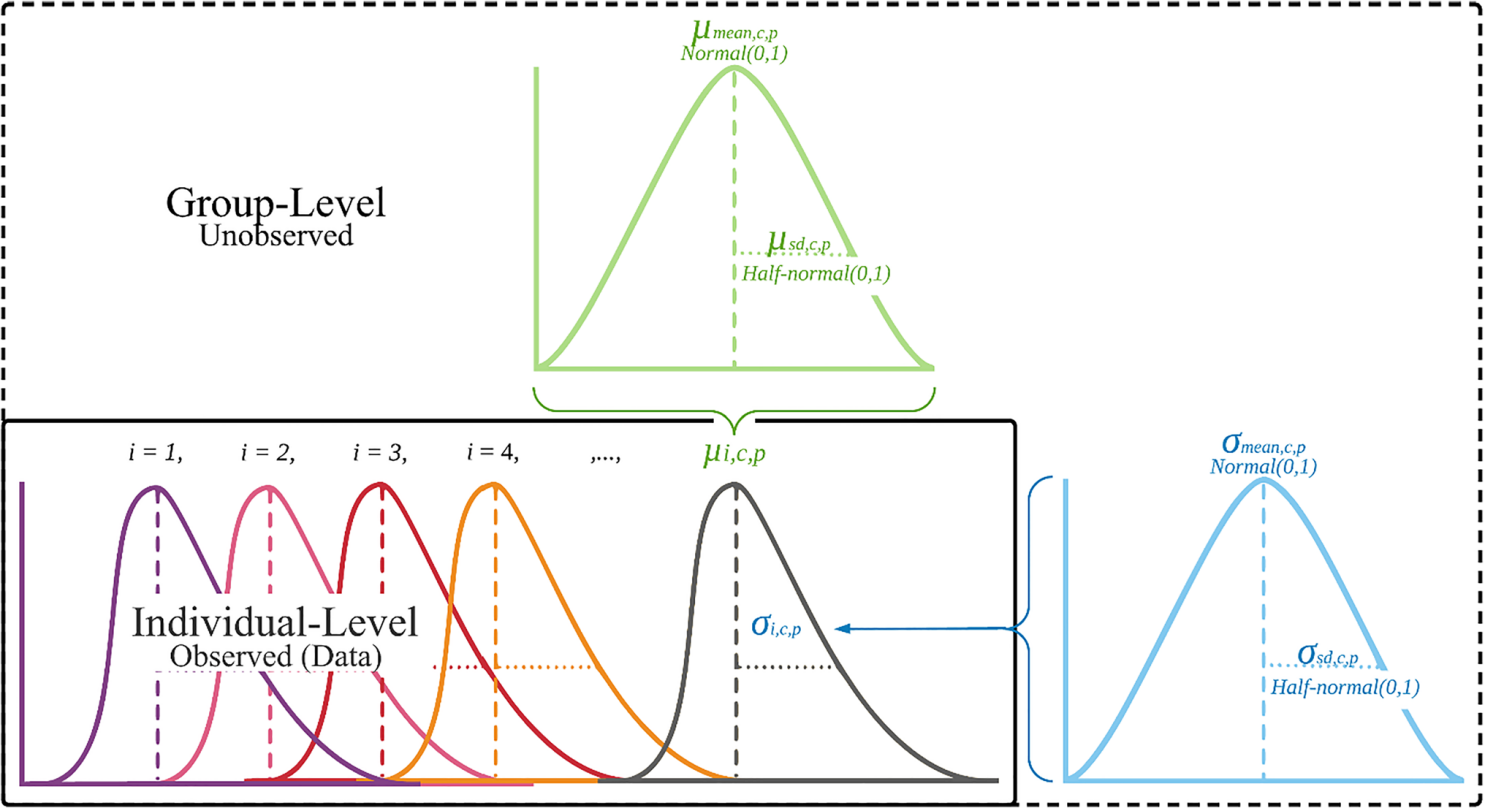
A structured schematic representation of the hierarchical model. *Note. i* = subject; *c* = condition; *p* = phase; *sd* = standard deviation; *μ*_*i*_ = individual-level mean parameter; *σ*_*i*_ = individual-level variability parameter

Furthermore, the individual-level means (i.e., *μ*_*i*, *c*, *p*_; referred to as mu) and standard deviations (i.e., *σ*_*i*, *c*, *p*_; referred to as sigma) were extracted for each condition and phase. All R scripts and the Stan model file are available on https://osf.io/pqvga/. A graphical representation of the model is included as well (see Fig. 2). The extracted delta, mu, and sigma parameters for each task and session combination are available on https://osf.io/pqvga/. All relevant convergence statistics have been extracted and are visually presented on https://osf.io/pqvga/ as well.

#### Between-measure correlations

For computation of the comprehensive between-task correlations that are reported in the Appendix, we utilized Spearman’s rho (*ρ*). In particular, Spearman’s rho (*ρ*) is a good non-parametric substitute for the parametric Pearson’s *r*, since Pearson’s *r* assumes that the relationship between two variables is both monotonic and linear (among other assumptions). The relationship between RT and error rate indices of cognitive-behavioral tasks is often monotonic, but not necessarily linear (Hedge, Powell, Bompas, et al., 2018a). Thus, Spearman’s rho will likely provide a more robust alternative, since Pearson’s *r* assumptions are not likely to be met. However, for the between-task and within-task analyses discussed in the Results section below, the focus was on *reaction time* indices associated with common difference score measures (e.g., RT Stroop effect). Hence, with the linearity assumption met, we employed Pearson *r* correlations for the latter hierarchical Bayesian within-, and between-task, correlational analyses.

## Results

### Reliability estimates: Traditional approach

Due to the large number of measures, all reliability estimates are presented in Appendix 1 (Appendix Tables 6, 7, 8, 9, 10 and 11). There, a full report includes internal consistency and test–retest reliabilities for the aggregate measures (mean RT, error rate) for all trial types, across all tasks and sessions. Although the aggregate measures are briefly discussed, only the difference score results are presented here due to their theoretical importance as measures of cognitive control, and within the DMCC battery (Tang et al., 2022). Table 1 presents both the split-half and test–retest reliability estimates for RT, computed separately for each control mode condition (baseline, reactive, proactive), for each task paradigm (3 x 3 x 4 = 36 estimates total). The corresponding 36 error rate estimates are shown in Table 2. In addition, for the AX-CPT task, four additional derived indices were also examined in addition to the difference scores (A-cue bias, d’-context, and Proactive Behavioral Index (PBI) for both RT and errors; see Table 3). These AX-CPT-derived estimates have been commonly employed as theoretically sensitive measures of cognitive control in this task, and have also been the focus of prior psychometric investigations (Boudewyn et al., 2015; Cohen et al., 1999; Lin et al., 2022; Richmond et al., 2015; Stawarczyk et al., 2014). Consequently, they were also of particular interest, to determine whether psychometric properties were improved within the context of the DMCC battery and experimental manipulations. For ease of interpretation, estimates of test–retest reliability below .50 are considered poor; between .50 and .75 are considered moderate; between .75 and .90 are considered good; and above .90 are considered excellent (Koo & Li, 2016). However, these thresholds are somewhat arbitrary; they are offered here as a guide. Of course, the qualitative description of reliability is not a substitute for understanding the numerical estimate in its context.

**Table 1:** Reaction time reliability across sessions

Measure	Split-half (95% CI)	ICC2,1 (95% CI)	ICC3,1 (95% CI)	M (ms)	Range (ms)
Baseline					
Stroop effect	.82 (.69−.90)	.27 (.11−.42)	.29 (.12−.44)	137	− 267 to 385
BX interference	.71 (.60−.80)	.45 (.26−.60)	.49 (.33−.61)	75	− 109 to 872
TRCE	.48 (.21−.67)	.30 (.13−.45)	.30 (.13−.45)	77	− 319 to 921
Recency effect	.08 (– .22−.38)	.26 (.04−.45)	.27 (.05−.47)	117	− 201 to 480
Proactive					
Stroop effect	.68 (.40−.84)	.34 (.18−.49)	.34 (.18−.49)	83	− 200 to 300
BX interference	.77 (.69−.84)	.50 (.34−.62)	.49 (.34−.62)	51	− 91 to 493
TRCE	.57 (.36−.72)	.36 (.20−.50)	.37 (.21−.51)	62	− 236 to 683
Recency effect	.29 ( .03−.51)	.34 (.14−.52)	.36 (.15−.54)	169	− 180 to 560
Reactive					
Stroop effect	.90 (.81−.95)	.30 (.14−.45)	.30 (.14−.45)	93	− 480 to 479
BX interference	.70 (.58−.78)	.50 (.35−.63)	.50 (.35−.63)	125	− 52 to 510
TRCE	.59 (.42−.72)	.40 (.25−.54)	.40 (.25−.54)	94	− 642 to 967
Recency effect	.19 (– .09 to .45)	.29 (.08−.48)	.31 (.09−.50)	85	− 176 to 350

*Note.* Split-half is an average of the test and retest phase split-half reliabilities. ICC2,1 is a two-way random effects, absolute agreement, single rater intraclass correlation coefficient; a measure of test–retest reliability. ICC3,1 is a two-way mixed effects, consistency, single rater intraclass correlation coefficient; a measure of test–retest reliability. *CI* confidence interval, *M* mean

**Table 2:** Error rate reliability across sessions

Measure	Split-half (95% CI)	ICC2,1 (95% CI)	ICC3,1 (95% CI)	M	Range
Baseline					
Stroop effect	.43 (.19−.61)	.27 (.10−.42)	.28 (.11−.43)	3.0%	– 5 to 26%
BX interference	.62 (.50−.72)	.27 (.06−.45)	.33 (.15−.48)	1.08	–.52 to 2.83
TRCE	.73 (.64−.80)	.16 (– .03−.33)	.16 (–.03−.33)	7.1%	– 12 to 56%
Recency effect	– .21 (– .42−.07)	.10 (– .10−.30)	.11 (–.12−.32)	13.8%	– 12 to 60%
Proactive					
Stroop effect	.48 (.16−.69)	.38 (.22−.52)	.38 (.22−.52)	1.7%	– 4 to 18%
BX interference	.62 (.50−.73)	.29 (.11−.45)	.29 (.11−.45)	.93	–.50 to 2.47
TRCE	.74 (.66−.80)	.46 ( .30−.60)	.46 ( .30−.60)	10.7%	– 14 to 56%
Recency effect	.01 (– .28−.31)	.04 (–.19−.26)	.04 (–.19−.26)	20.6%	– 25 to 60%
Reactive					
Stroop effect	.88 (.84−.92)	.79 (.71−.85)	.79 (.71−.85)	2.3%	– 28 to 21%
BX interference	.72 (.62−.80)	.41 (.20−.57)	.45 (.29−.59)	.93	–.27 to 3.18
TRCE	.80 (.73−.86)	.18 (–.01−.35)	.18 (–.01−.35)	5.1%	– 11 to 54%
Recency effect	.48 (.24−.66)	.24 (.03−.43)	.26 (.03−.45)	8.3%	– 25 to 50%

*Note.* Split-half is an average of the test and retest phase split-half reliabilities. ICC2,1 is a two-way random effects, absolute agreement, single rater intraclass correlation coefficient; a measure of test–retest reliability. ICC3,1 is a two-way mixed effects, consistency, single rater intraclass correlation coefficient; a measure of test–retest reliability. *CI* confidence interval, *M* mean

**Table 3:** AX-CPT-derived indices reliability across sessions

Measure	Split-half (95% CI)	ICC2,1 (95% CI)	ICC3,1 (95% CI)	M	Range
Baseline					
A-cue bias	.55 (.41−.66)	.23 (.05−.39)	.23 (.05−.39)	.09	– 1.14 to .87
*d′* context	.78 (.70−.84)	.39 (.17−.56)	.45 (.28−.58)	2.85	– .23 to 4.4
PBI_error_	.69 (.59−.76)	.15 (– .03 to .32)	.18 (– .03 to .37)	– .18	– .94 to .89
PBI_rt_	.72 (.62−.80)	.26 (.05−.44)	.32 (.15−.48)	.03	– .40 to .24
Proactive					
A-cue bias	.79 (.71−.85)	.42 (.24−.57)	.42 (.24−.57)	.37	– 1.99 to 1.47
*d′* context	.80 (.72−.85)	.55 (.40−.66)	.55 (.40−.66)	3.09	– .92 to 4.40
PBI_error_	.80 (.73−.86)	.39 (.20−.56)	.39 (.19−.56)	.16	– .89 to .94
PBI_rt_	.80 (.72−.85)	.51 (.36−.64)	.51 (.36−.64)	.09	– .26 to.32
Reactive					
A-cue bias	.53 (.39−.64)	.33 (.16−.48)	.33 (.16−.48)	.06	– .80 to.82
*d′* context	.79 (.72−.85)	.56 (.40−.68)	.58 (.45−.69)	2.93	.58 to 4.4
PBI_error_	.65 (.54−.74)	.20 (.00−.38)	.22 (.01−.41)	– .09	– .93 to .86
PBI_rt_	.59 (.46−.70)	.43 (.27−.57)	.43 (.27−.57)	.02	– .30 to .21

*Note.* Split-half is an average of the test and retest phase split-half reliabilities. ICC2,1 is a two-way random effects, absolute agreement, single rater intraclass correlation coefficient; a measure of test–retest reliability. ICC3,1 is a two-way mixed effects, consistency, single rater intraclass correlation coefficient; a measure of test–retest reliability. *CI* confidence interval, *M* mean

As expected, the reliabilities of difference score measures were weaker than the reliabilities of aggregate measures. For example, the split-half reliability for Stroop incongruent RT was on average *r* = .99 across sessions, Stroop congruent RT was on average *r* = 1.00 across sessions (see Appendix 1), but the reliability of the RT Stroop effect was on average *r* = .82 across sessions. The same general pattern is observed for the test–retest reliability RT estimates: *r* = {.79, .93, .43}, respectively. This pattern is observed across all tasks, for both split-half and test–retest reliability estimates, for both RT and accuracy measures.

For the Stroop, cued task-switching, and AX-CPT difference score estimates the reliability results yield mixed conclusions. The split-half estimates indicate mostly moderate-to-good reliability, for both RT and error rate (x̅ = .68, range = .43–.90). However, the test–retest estimates indicate poor reliability, regardless of which ICC computation was used, ICC2,1: x̅ = .40, range = .16–.79; ICC3,1: x̅ = .42, range = .16–.98. Moreover, the session level manipulations (i.e., proactive and reactive variants) did not produce demonstrative improvements in reliability. Although reliability was generally highest in the reactive session, the overlapping confidence intervals across sessions suggests that this was not a robust effect.

The reliability of the AX-CPT-derived indices revealed a similar pattern as the difference score measures; the split-half reliability estimates were stronger than test–retest estimates. In contrast, two novel and interesting patterns emerged. First, all four proactive session-derived indices were internally consistent, with split-half estimates ranging from .79–.80. Second, split-half estimates for *d′-context* exceeded the reliability threshold of .75 in all sessions and thus is considered to be internally consistent as well. This suggests that the reliability of the *d′-context* and the proactive indices should not pose a bottleneck when used to examine between-measure correlations.

In the Sternberg task, the recency effect measure was found to be generally unreliable, in both RT and error rate. The poor reliability and high variability of the Sternberg estimates may stem from the task design (i.e., low number of observations available to calculate a difference score). To induce proactive control, recent negative (RN) trials were presented infrequently in the baseline and proactive sessions, with only eight RN trials per subject. It is therefore not advised to calculate a traditional difference score from the current Sternberg paradigm for use in individual differences research.

Overall, the reliability analyses computed in the traditional manner suggested that the DMCC cognitive control tasks were not robust, particularly for test–retest reliability, a finding consistent with that of other psychometric analyses of cognitive control tasks (Hedge, Powell, & Sumner, 2018b; Kucina et al., 2022; Rey-Mermet et al., 2018; Rouder & Haaf, 2019). That said, the difference between split-half and test–retest estimates of reliability is intriguing and may provide some insight into the measurement of cognitive control; we discuss this finding in more detail in the discussion section. We next examined whether the reliability analyses produce different results when computed using HBM approaches to estimation.

### Reliability estimates: Hierarchical Bayesian modeling approach

**Fig. 3:**
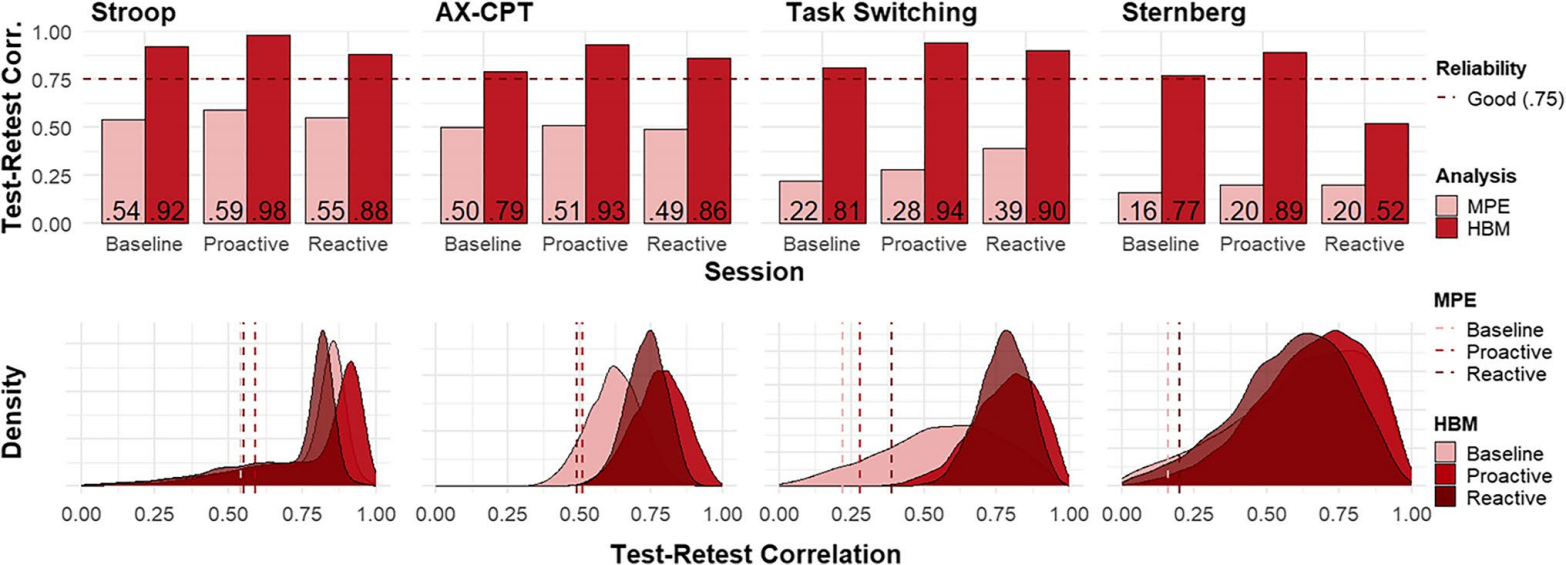
Test–retest reliability estimates of the difference score parameter. *Note.* Distribution of observed reliability estimates, split by analysis type for comparison. Density plot to visualize uncertainty of HBM delta estimate, dashed line of respective MPE estimates for comparison of reliability magnitude. *MPE* Pearson correlation coefficient obtained from traditional mean point estimates approach; *HBM* Pearson correlation coefficient of delta estimates obtained by hierarchical Bayesian modeling. *n* ranges between 104 and 122; different *n* sample sizes due to additional multivariate outlier removal

As shown in the first set of analyses, we were not able to extract reliable individual differences from experimental task difference score measures. The goal of the second set of analyses was to examine whether hierarchical Bayesian modeling improved reliability estimation in the DMCC task battery data. This also replicates the modeling approach of prior work similar to the current study (i.e., Haines et al., 2020; Rouder & Haaf, 2019). For a comparison between the traditional MPE and HBM approach, the corresponding mean point-estimate of test–retest reliability (also using Pearson *r* to increase comparability) is provided as well in Fig. 3. As guidelines for test–retest reliability, we again follow Koo and Li’s (2016) thresholds (i.e., respectively, poor, moderate, good, excellent : < .50, .50–.75, .75–.90, > .90). Although those guidelines are for ICC, commonly accepted test–retest correlation guidelines based on Pearson’s product-moment correlation coefficient do not exist to our knowledge.

In contrast to the traditional psychometric approach to estimating test–retest reliability (i.e., based on mean point estimates), which indicated poor-to-moderate test–retest reliability (x̅_r_ = .39), the HBM extracted estimates of test–retest reliability could be classified as good to excellent (all above .75, x̅_r_ = .85), with the only exception being the Sternberg recency effect in the reactive condition (*r* = .52). The strong reliability estimates obtained using the HBM approach are consistent with Haines et al. (2020), and Rouder and Haaf (2019). The test–retest estimates of the delta parameter indicate that HBM can indeed provide reliable individual differences from cognitive control tasks, even when using a difference score index^3^. An additional interesting pattern emerged when comparing test–retest reliability in the different control mode conditions. In particular, reliability was highest for the proactive conditions (x̅ = .94; vs. x̅ = .82 for baseline, and x̅ = .79 for reactive), which also differed from the pattern observed in the traditional ICC analyses (for which the reactive condition tended to show the highest values).

### Between-measure correlations

#### Within-task correlations

**Fig. 4:**
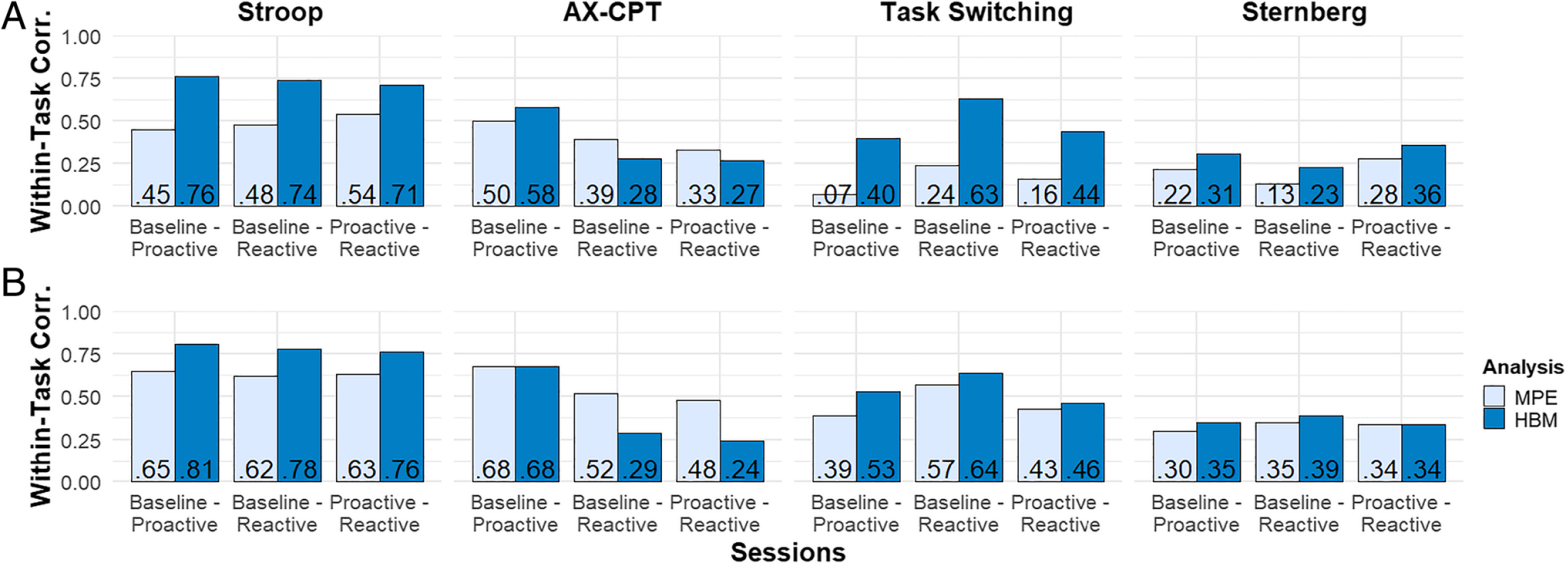
Within-task correlation estimates of the difference score parameter. *Note.* Distribution of observed correlations within task paradigms, split by analysis type for comparison. MPE = Pearson correlation coefficient obtained from traditional mean point estimates approach; HBM = Pearson correlation coefficient of delta estimates obtained by hierarchical Bayesian modeling. *n* = 116. **a** Correlations calculated on test (i.e., time 1) data only. **b** Correlations calculated on combined test and retest (i.e., time 1 & 2) data

Next, our analyses examined the correlations between measures while comparing the traditional MPE estimates to the HBM-derived ones. We began by focusing on correlations within the same task paradigms, between sessions (see Fig. 4). Because these are within-task correlations, we expected them to be consistently positive and overall relatively high, since the experimental manipulations of cognitive control mode are quite subtle. Thus, they provided a potentially more useful testbed from which to examine the relationship between reliability of measures and their correlations.

Because of the potential for learning effects that might impact within-task correlations, we first conducted these analyses restricted to cognitive control estimates from the test phase only (see Fig. 4a). The average within-task correlations derived with the MPE approach were weak to moderate (x̅ = .32), with a maximum correlation (between Stroop proactive and reactive) of *r* = .54. In comparison, the values of the HBM-derived correlations were on average moderate (x̅ = .48), with a maximum correlation (between Stroop baseline and proactive) of *r* = .76. Although the test phase correlations are overall lower than expected, it is particularly true for the MPE task switching and MPE Sternberg estimates. However, a clear pattern did emerge: with the exception of two AX-CPT correlations, the HBM estimates are higher than their MPE counterparts. Indeed, a Wilcoxon signed-rank test with continuity correction suggests the difference in the strength of the HBM relative to the MPE correlations was significant at α = .05 (p = .01).

##### Reliability and within-task correlations

**Fig. 5:**
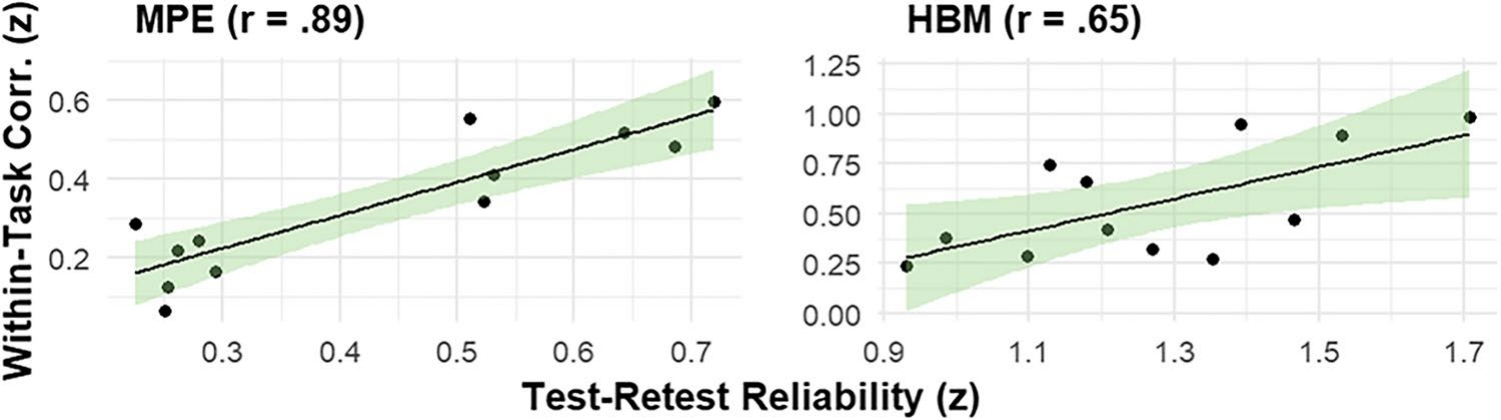
Standardized relationship between reliability and within-task correlations. *Note.* MPE = Pearson correlation coefficient obtained from traditional mean point estimates approach; HBM = Pearson correlation coefficient of delta estimates obtained by hierarchical Bayesian modeling; *n* = 104. Both reliability and within-task correlations were r-to-z transformed. Green area is 95% confidence interval around linear regression line

We then examined the relationship between the test–retest reliabilities and within-task correlations. In particular, we experimentally tested the key psychometric principle that reliability serves as a bottleneck to individual differences analysis. To test this principle, we examined whether the strength of within-task correlations was related to the level of reliability in the estimates. For these analyses, we used Pearson correlation as an indicator of pattern similarity between the two measures (values near +1 reflect high similarity, values near 0 reflect low similarity), after first computed r-to-z transformations to linearize the within-task correlation values. Indeed, the results were supportive of the hypothesis (see Fig. 5). For the MPE-derived estimates, the distribution of test–retest reliabilities exhibited a highly similar pattern to the distribution of within-task correlations (*r* = .89); a similar relationship was found for the HBM-derived estimates (*r* = .65). Thus, when examining the within-task relationships, we find clear support for the hypothesis that the higher within-task correlations that we observed in the HBM extracted values was closely related to their overall higher reliabilities.

#### Re-analyses with combined test and retest data

As a follow-up, we conducted the latter two analyses again, but now combining the test and retest data to observe the impact of data aggregation. Interestingly, a different pattern emerged (see Fig. 4b). The within-task correlations derived with the MPE approach increased strongly to a moderate level (x̅ = .50 versus x̅ = .38 with test-phase only data), with a maximum correlation (between AX-CPT baseline and proactive) of *r* = .68. In comparison, the values of the HBM-derived correlations were quite similar (x̅ = .52 versus x̅ = .48 with test-phase only data), with a maximum correlation (between Stroop baseline and proactive) of *r* = .81. Unlike with the test phase only data, here a Wilcoxon signed-rank test with continuity correction suggested *no* significant difference between test + retest MPE and HBM estimates at α = .05 (*p* = .41). Nevertheless, the same relationships were observed between reliability level and the strength of within-task correlations (MPE: *r* = .82; HBM: *r* = .61). Taken together, these findings are consistent with prior research that suggests HBM-derived estimates are more stable and robust than the traditional MPE approach, particularly when fewer trials are available for estimation (Efron & Morris, 1977; Hox et al., 2012; Smid et al., 2020). But in all cases, the data are strongly supportive of the psychometric principle that tasks with lower reliabilities will tend to be associated with reduced strength in individual differences correlations.

#### Between-task correlations

Lastly, we conducted a more comprehensive examination of between-task correlations in the DMCC battery, first using the traditional MPE estimates. Because of the large number of tasks, conditions, and measures, we relegate full reporting of these correlations to Appendix 2, and only provide a brief summary here. In total, we examined 198 between-task correlations with a median correlation of *r* = .13, and of these only 12 had an absolute magnitude of *r* = .25. These values are on par with the so-called “crud factor” in differential psychology, which refers to the idea that correlations with magnitudes between 0 and .20 should be interpreted as nothing but noise (Lykken, 1968; Meehl, 1986; but see Orben & Lakens, 2020 for a recent critique).

We then focused on between-task, same-condition correlations (e.g., correlation of Stroop baseline to AX-CPT baseline) of key difference score measures and compared between traditional MPE and HBM approaches. Examining both approaches on the test phase only data (see Table 4), reveals that 33 out of 36 correlations are between *r* = – .20 and *r* = .20, with the remaining 3 correlations not being much higher (*r* = {– .23, .23, .24}). Following the analyses of the within-task correlations, we also calculated the between-task correlations on both test and retest phase data. A similar pattern emerged: 34 out of 36 correlations are between *r* = – .20 and *r* = .20, with the remaining two correlations again not being much higher (*r* = {– .21, .27}). Moreover, there was no consistent difference between the correlations computed from the traditional MPE (x̅ = .03) and HBM estimated values (x̅ = – .01) at test only, nor for test plus retest phase data (x̅ = .03), (x̅ = – .01), respectively. Thus, the results of this analysis do not support our hypothesis that the increased test–retest reliabilities observed in the HBM parameters would also translate into higher between-task correlations.

**Table 4:** Between-task correlations of test phase reaction time difference score parameters

Session	Index 1	Index 2	MPE	HBM	*n*
Baseline	Stroop effect	BX interference	.11	– .20	87
Baseline		TRCE	.13	.05	87
Baseline		Recency effect	.15	.01	87
Baseline	BX interference	TRCE	.03	– .03	87
Baseline		Recency effect	– .03	.07	87
Baseline	TRCE	Recency effect	– .10	– .05	87
Proactive	Stroop effect	BX interference	.11	.00	75
Proactive		TRCE	– .02	.04	75
Proactive		Recency effect	– .04	– .06	75
Proactive	BX interference	TRCE	.03	– .04	75
Proactive		Recency effect	– .23	– .20	75
Proactive	TRCE	Recency effect	.05	.07	75
Reactive	Stroop effect	BX interference	.23	.24	102
Reactive		TRCE	.18	.15	102
Reactive		Recency effect	– .03	– .15	102
Reactive	BX interference	TRCE	.07	.07	102
Reactive		Recency effect	– .10	– .16	102
Reactive	TRCE	Recency effect	.01	– .05	102

*Note.* Indices are based on test phase only. *MPE* Pearson *r* correlation of mean point estimated differences scores; *HBM* Pearson *r* correlation of hierarchical Bayesian modeling estimated differences scores; *TRCE* task-rule congruency effect. Variability in sample sizes due to between-task differences in pre-processing

**Table 5:** Between-task correlations of test + retest phase reaction time difference score parameters

Session	Index 1	Index 2	MPE	HBM	*n*
Baseline	Stroop effect	BX interference	.17	.13	87
Baseline		TRCE	– .10	– .01	87
Baseline		Recency effect	.11	– .02	87
Baseline	BX interference	TRCE	.12	.03	87
Baseline		Recency effect	.03	– .18	87
Baseline	TRCE	Recency effect	– .03	– .01	87
Proactive	Stroop effect	BX interference	.11	.05	75
Proactive		TRCE	.27	.10	75
Proactive		Recency effect	.10	– .05	75
Proactive	BX interference	TRCE	– .14	– .09	75
Proactive		Recency effect	.14	– .00	75
Proactive	TRCE	Recency effect	– .19	– .21	75
Reactive	Stroop effect	BX interference	.15	.11	102
Reactive		TRCE	– .09	– .06	102
Reactive		Recency effect	– .03	– .03	102
Reactive	BX interference	TRCE	– .11	– .10	102
Reactive		Recency effect	.15	.20	102
Reactive	TRCE	Recency effect	– .15	– .04	102

*Note.* Indices are based on averaged test and retest phases. *MPE* Pearson *r* correlation of mean point estimated differences scores; *HBM* Pearson *r* correlation of hierarchical Bayesian modeling estimated differences scores; *TRCE* task-rule congruency effect. Variability in sample sizes due to between-task differences in pre-processing

## Discussion

The goal of the current study was to examine psychometric reliability in experimental tasks of cognitive control. To this end, we utilized the new DMCC task-battery, as it comprised classic cognitive control tasks, but also included theoretically derived task variants that could isolate effects related to engagement of proactive and reactive control modes (Braver, 2012; Braver et al., 2021). It was our primary hypothesis that psychometric reliability would not be satisfactory in these tasks, when using traditional summary-statistic approaches, but that a different conclusion would be drawn when reliability was estimated with HBM approaches, which are likely to better capture individual differences variability associated with task performance in this domain. Indeed, when using traditional statistical approaches (i.e., split-half, ICC), the psychometric analyses suggested that our theoretically optimized task battery did not improve reliability above and beyond that of existing tasks and batteries. Plainly stated, the reliability of the DMCC task battery measures, when computed with popular difference score indices, were moderate at best, which is quite consistent with prior psychometric reports using different task variants (von Bastian et al., 2020 see also; Hedge, Powell, & Sumner, 2018b; Rouder & Haaf, 2019). In particular, when analyses were conducted with traditional psychometric methods, there was no evidence suggesting improved reliability associated with metrics of proactive and reactive cognitive control.

One important finding was that, with the conventional analyses, reliability estimates focused on internal consistency (i.e., split-half indices) were almost always higher than those focused on temporal stability (i.e., test–retest; i.e., ICC2,1 & ICC3,1). Given that split-half methods are calculated on a single timepoint measure, and test–retest on two (or more) timepoint measures, this finding is not surprising. It does, however, reaffirm that the two methods cannot be treated as interchangeable indices of reliability. When possible, an index of both internal consistency and temporal stability should be reported. Importantly, the observed discrepancy indicates that our measures of cognitive control have some internal consistency, but additional work needs to be conducted to determine why temporal stability appears to be lower than desirable. In our case, the “additional work” meant that we investigated whether traditional psychometric statistics might not be appropriate or well aligned for the calculation of individual differences in experimental cognitive control tasks.

In particular, we utilized hierarchical Bayesian modeling (HBM) as an alternative approach, to provide another test of the hypothesis suggested from recent work, that this approach might be better suited for reliability estimation with cognitive experimental tasks (Haines et al., 2020; Rouder & Haaf, 2019). Our results were strongly consistent with this hypothesis. Specifically, we found that with HBM estimation, the cognitive control indices were actually highly reliable, even when using indices derived from difference scores. Specifically, our findings indicate that test–retest reliability estimates for the delta (difference score) parameters in our sample can be almost always classified as good, and sometimes even excellent. This finding is a striking one, particularly when compared to the weak and moderate intraclass correlation coefficients (ICC) observed in the traditional set of analyses. The HBM analyses clearly suggest that accounting for individual-level variability and the type and shape of the distribution can “rescue” the reliability estimation, using the formulation of Rouder and Haaf (2019). Interestingly, it was also found that in both the traditional and HBM analyses, reliability estimates were highest for the proactive task variants, which also supports our hypothesis that theoretical motivated task manipulations may contribute to improved reliability.

One of the primary reasons for the enduring importance and need for attention to reliability measures is the view – which is well accepted in the psychometric literature (Hedge, Powell, & Sumner, 2018b; Parsons et al., 2019; Rouder et al., 2019; Spearman, 1904) – that reliability might serve as a bottle-neck or constraint on the ability to detect correlations between measures of individual differences. The key point is that, for measures with low reliability, there should be reduced sensitivity for the detection of between-measure correlations. Yet this assumption has been rarely experimentally tested (Cooper et al., 2017).

Our analyses also provided experimental support for this contention, when examining correlations between DMCC task measures within tasks (i.e., between control modes; baseline, proactive, reactive). Specifically, we assumed that within-task correlations could be treated as “benchmarks” since we assumed ground-truth positive correlations, given that the same subjects were performing subtle variants of the same task across sessions. Indeed, we found that not only was test–retest reliability increased with HBM estimates relative to the traditional ICC measures, but also so were the within-task correlations. Thus, the results provide clear support for the psychometric perspective, in demonstrating the importance of reliability, as well as the improved potential to estimate individual differences in cognitive control with HBM-based approaches. Moreover, we conducted analyses that compared results when using test-phase only data, which we assumed would be less impacted by learning or strategy effects, with those combining the test and retest data together. This comparison also revealed the advantages of the HBM approach, as the within-task correlation strengths were more stable across both sets of analyses; conversely, with the MPE estimates, the strength of within-task correlations was quite lower when using the test-phase only data. This suggests that MPE-based estimates of reliability and correlation will be more susceptible and impacted by the amount of data available for analysis, whereas HBM estimates are more stable and efficient.

Unfortunately, the one dimension to which the increased reliability obtained with HBM-estimates *did not* translate into improved correlation strength, was in the correlations observed between DMCC tasks. Here, we observed on average near-zero correlations (with majority less than *r =* 0.2) that did not differ from difference score measures derived with a traditional approach, nor between test only and test plus retest data. Thus, at least in the case of the DMCC task battery, it cannot be claimed that the weak between-task correlations are due to the unreliability of the measures.

Indeed, the contrast among the within-task and between-task correlations is striking. Moreover, it clearly points to the need for future research to understand the basis for the repeated findings of low between-task correlations among cognitive control measures (von Bastian et al., 2020), particularly given that our results argue against an interpretation in terms of low measurement reliability. As such our findings converge strongly with that of Rouder and Haaf (2019), who also observed that HBM estimates were associated with increased test–retest reliability in cognitive control tasks but did not change the nature of cross-task correlations. We discuss this issue further below, along with other limitations of the current work and fruitful directions for further research.

## Limitations and future directions

The current study design, though promising as validation of the newly developed DMCC task battery in terms of its psychometric robustness, does come with some limitations. First, it is important to acknowledge the fully online format of the design. This design has clear and significant advantages, the foremost of which is that the multi-session nature of the study would place a stronger burden on subjects if frequent laboratory visits were required. Moreover, at the time of this writing, the SARS-CoV-2 pandemic has accelerated this shift of experimental research towards an online format. Finally, much work has validated online task administration as a viable format for cognitive tasks, with many important results replicated (Anwyl-Irvine et al., 2021; Bridges et al., 2020; Chaytor et al., 2021; Crump et al., 2013; Pronk et al., 2021). Nevertheless, the online format also has a number of drawbacks, which are also well known in the literature. These include reduced experimental control over the task environment, and an increased risk of potential distractions being present.

Another limitation of the design comes from the fact that not all the tasks were optimized to be delivered in a test–retest format. In addition to standard concerns about practice effects impacting retest sessions, the DMCC battery also includes some tasks and conditions that are likely to be more impacted by prior experience than others. For example, in the Cued-TS proactive and reactive conditions, incentives are given based on performance, although these are not present in the baseline condition. During the initial baseline condition, subjects are not told about the potential for incentives in the subsequent proactive and reactive sessions. However, during the retest baseline session, they do have this knowledge, which could impact the cognitive strategies used in this session. Likewise, in the AX-CPT proactive condition, subjects receive explicit strategy training for how to utilize the contextual cues. Again, in the preceding baseline test session, which is otherwise identical to proactive, they have not yet received this strategy training, but in the retest baseline sessions subjects have already had much experience in following the strategy instructions, which could also impact their performance in this session. Thus, in future investigations of test–retest reliability with the DMCC battery, it would be useful to reconsider the manipulations used for the proactive and reactive sessions, to minimize the carry-over effects of prior practice. Conversely, however, we found that at least for HBM estimates, the strength of within-task correlations was not strongly impacted by whether test-phase only or both the test and retest data were combined. This suggests that such concerns may primarily impact traditional analyses based on MPE estimates, for which the results may be more strongly impacted by both the amount and variability present in the within-subject data.

The current study adds to a growing literature highlighting the promise and potential of HBM approaches for analyzing cognitive experimental tasks. Yet, currently these types of Bayesian analyses are still relatively rare in the literature; consequently, there is still a poor understanding of how they are different from traditional analyses, or how effects might diverge. Given the lack of widespread adoption of HBM methods, we opted for a more conservative approach, of first presenting results from traditional psychometric analyses of reliability, before comparing them with HBM estimates. We utilized Bayesian models that estimated effects for each task-variant separately, following current literature (Haines et al., 2020; Rouder & Haaf, 2019).

However, the approach can be expanded to a single all-encompassing model. In particular, it is also possible to develop a generative model in which the different conditions and even different tasks are assumed to be additional level(s) of hierarchy from which the distributions arise (i.e., analogous to the way subjects are drawn from a higher-level distribution) (see also Rouder et al., 2019 (in pre-print)). Our current model benefits from shared information across subjects and trial-types (i.e., congruent, incongruent), but only within one variant (i.e., baseline, proactive, reactive) of each task-paradigm. A complete generative model has the benefit of between-condition and between-task information sharing as well. However, building full generative models will increase the complexity of the modeling endeavor, so it is worthwhile to progress in a more incremental fashion. Nevertheless, the promise of the current approach suggests that further development of Bayesian statistical approaches to task parameter estimation may be a particularly worthwhile direction for the field (Gelman et al., 2013; Lee & Wagenmakers, 2014; McElreath, 2020).

As part of the limitations of the current study, we acknowledge recent work suggesting that analyses solely based on reaction time measures also pose a challenge in interpreting results. For example, Draheim et al. (2019, 2020) have argued that the use of reaction time difference scores “is the primary cause of null and conflicting results” when examining individual differences in attentional control. Their work suggests that measures based on accuracy rather than reaction time can improve reliability, intercorrelations among tasks, latent factor scores, and associations with measures of working memory and fluid intelligence. Although it was beyond the scope of the current study, it is of course possible to use HBM approaches with accuracy measures as well, which suggests another possible direction for future work (Lin et al., 2022). Other work by Hedge et al. (2021) suggests the importance of cognitive modeling to properly estimate latent processes, for example by employing a diffusion model for conflict tasks (Rey-Mermet et al., 2021; Ulrich et al., 2015; Weigard et al., 2021). In this work it was found that, when conflict processes were decomposed from non-conflict processes, only weak correlations (*r* < .05) were observed between conflict processes across different cognitive control tasks. Contrarily, correlations between model parameters representing processing speed and strategy were consistently positive, with moderate to strong correlations. Future work should follow suit and use cognitive models that account for the speed–accuracy tradeoff and the multiple latent processes that underlie observed measures.

The key unresolved question from the current study relates to the low between-task correlations observed, even among the theoretically derived tasks that comprise the DMCC battery. These findings are not unprecedented; indeed, they are quite consistent with a number of prior studies that have examined correlations among cognitive control measures through task batteries and latent variable modeling (Draheim et al., 2020; Rey-Mermet et al., 2018; Rouder & Haaf, 2019; von Bastian et al., 2020). Nevertheless, the current results are quite discouraging, as they increase doubt on the domain-generality of cognitive control constructs. In some ways, however, the results are discrepant from work that has been emerging from the neuroimaging literature, which has also become more attuned to questions of individual differences and domain-generality (Dubois & Adolphs, 2016; Elliott et al., 2020; Finn et al., 2017; Freund et al., 2021; Gratton et al., 2018).

Indeed, within the neuroimaging literature, an important emerging finding is that although lower-dimensional (e.g., “univariate”) descriptions may not be reliable for characterizing individual differences in brain activity, higher-dimensional (e.g., multivariate) descriptions can be quite discriminative. This can be seen most clearly in “fingerprinting” studies (Finn et al., 2015), in which pattern similarity techniques demonstrate that individuals show high test–retest reliability, such that their activation profile from a test scan can be easily discriminated from other individuals in a retest session (i.e., significantly higher test–retest similarity within-individuals than between). Moreover, our group has extended this approach into the domain of task fMRI and cognitive control, using twin-based study designs to demonstrate a remarkable degree of similarity among identical twin-pairs relative to unrelated pairs (or even fraternal pairs) in the fronto-parietal regions most strongly associated with cognitive control functions (Tang et al., 2021). Most strikingly, these effects were only observed when utilizing multivariate activation pattern similarity, rather than univariate measures (Etzel et al., 2020), and demonstrated clear domain-generality (i.e., cross-task effects; (Tang et al., 2021)). Together, this work suggests the possibility that utilizing multivariate rather than univariate descriptions of the individual might be a promising direction even for behavioral characterizations. Indeed, initial work in this direction, utilizing behavioral fingerprinting approaches, has begun (see Han & Adolphs, 2020), though much more investigation is needed.

## Conclusions

We examined whether well-established experimental tasks, but modified with theoretically aligned variants and task manipulations, are viable tools for measuring individual differences in cognitive control. As previously reported (Tang et al., 2022), the experimental manipulations included in this task battery were validated to be highly robust at the group level, in inducing consistent shifts towards proactive and reactive control. Yet, traditional psychometric approaches suggested that the theoretically derived cognitive control indices were not highly reliable, either in terms of internal consistency (split-half) or temporal stability (test–retest) measures, which were observed to be moderate at best. In contrast, when the test–retest data were re-examined using hierarchical Bayesian modeling, the findings were quite different, with good to excellent reliability observed in most measures. Moreover, these reliability effects translated into improved strength of within-task correlations.

Nevertheless, even with the reliable Bayesian estimates, between-task correlations were unaffected and remained uniformly poor, in other words, the poor between-task correlations were not due to reliability constraints. Together, these findings add to the growing literature suggesting the importance of Bayesian generative models when estimating individual differences, and its superior robustness to changes in number of observations when compared to traditional methods. Most importantly however, our findings also point to the need for further investigation into the source of low between-task correlations among experimental tasks that attempt to measure putatively domain-general cognitive control constructs. We encourage other researchers interested in cognitive individual differences to attend more closely to psychometric issues when conducting this important research.

## Figures and Tables

**Fig. 6:**
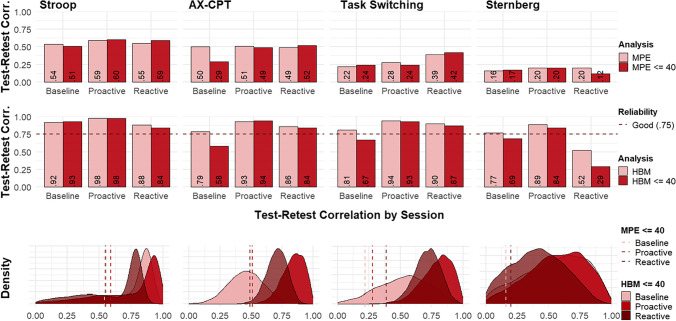
Test–retest reliability estimates of the difference score parameter. *Note.* Distribution of observed reliability estimates, split by analysis type for comparison. Density plot to visualize uncertainty of HBM delta estimate for the subset of subjects aged 40 and below, dashed line of respective MPE estimates for comparison of reliability magnitude for the same subset. MPE = Pearson correlation coefficient obtained from traditional mean point estimates approach; HBM = Pearson correlation coefficient of delta estimates obtained by hierarchical Bayesian modeling; MPE/HBM <= 40 = same analyses as their respective statistical approach, but on aged 40 and under subset. For MPE/HBM, *n* ranges between 104 and 122 and for MPE/HBM <= 40, *n* ranges between 71 and 89; different *n* sample sizes due to additional multivariate outlier removal

## Data Availability

The datasets generated during and/or analyzed during the current study are available in the Snijder et al., 2022 repository, https://osf.io/pqvga/

## Appendix 1

Appendix Tables 6, 7, 8, 9, 10 and 11

**Table 6:** Stroop (biased) reliability across sessions

Measure	Split-half (95% CI)	Test–retest (95% CI)	M	Range	Skew	Kurtosis
Baseline						
Reaction time						
Congruent	1.0 (1.0−1.0)	.92 (.88−.94)	749 ms	431–2706 ms	3.25	14.6
Incongruent	.99 (.99−1.0)	.94 (.90−.96)	918 ms	477–2851 ms	2.81	11.9
Stroop effect	.82 (.69−.90)	.27 (.11−.42)	137 ms	– 267 to 385 ms	– 1.00	6.71
Error						
Congruent	.93 (.88−.96)	.17 (.00−.32)	2.2%	0–24%	2.83	10.7
Incongruent	.79 (.71−.86)	.24 (.07−.39)	5.2%	0–40%	2.38	9.47
Stroop effect	.43 (.19−.61)	.27 (.10−.42)	3.0%	– 5 to 26%	1.78	4.71
Proactive						
Reaction time						
Congruent	.99 (.98−1.0)	.84 (.78−.88)	798 ms	415–3387 ms	3.45	18.2
Incongruent	1.0 (1.0−1.0)	.87 (.82−.91)	880 ms	450–3596 ms	2.95	13.2
Stroop effect	.68 (.40−.84)	.34 (.18−.49)	83 ms	– 200 to 300 ms	– .64	4.47
Error						
Congruent	.80 (.65−.89)	.70 (.59−.78)	1.2%	0–27%	4.88	32.8
Incongruent	.92 (.87−.94)	.80 (.72−.86)	2.9%	0–29%	3.38	16.2
Stroop effect	.48 (.16−.69)	.38 (.22−.52)	1.7%	– 4 to 18%	2.58	11.0
Reactive						
Reaction time						
Congruent	1.0 (1.0−1.0)	.87 (.82−.91)	790 ms	428–3787 ms	3.03	13.1
Incongruent	1.0 (1.0−1.0)	.84 (.78−.89)	882 ms	451–3763 ms	2.84	11.9
Stroop effect	.90 (.81−.95)	.30 (.14−.45)	93 ms	– 480 to 479 ms	– .27	15.8
Error						
Congruent	.98 (.96−.99)	.81 (.73−.86)	1.6%	0–40%	5.59	37.5
Incongruent	.90 (.86−.93)	.53 (.39−.64)	3.9%	0–42%	2.26	9.03
Stroop effect	.88 (.84−.92)	.79 (.71−.85)	2.3%	– 28 to 21%	– 2.03	15.2

*Note. N* = 126. *CI* confidence interval. Split-half is an average of the test and retest phase split-half reliabilities. Test–retest = ICC (2,1)

**Table 7:** Cued task switching (non-incentivized) reliability across sessions

Measure	Split-half (95% CI)	Test–retest (95% CI)	M	Range	Skew	Kurtosis
Baseline						
Reaction time						
Congruent	.99 (.98−.99)	.63 (.35−.78)	906 ms	448–2370 ms	2.48	12.70
Incongruent	.90 (.85−.94)	.52 (.36−.65)	983 ms	458–2657 ms	2.31	11.90
TRCE	.48 (.21−.67)	.30 (.13−.45)	77 ms	– 319 to 921 ms	1.70	7.00
Error						
Congruent	.89 (.86−.92)	.41 (.23−.55)	3.9%	0–38%	2.72	12.30
Incongruent	.84 (.80−.88)	.21 (.02−.38)	11%	0–60%	1.58	6.08
TRCE	.73 (.64−.80)	.16 (– .03 to.33)	7.1%	– 12 to 56%	1.19	2.10
Proactive						
Reaction time						
Congruent	.99 (.98−.99)	.79 (.67−.86)	718 ms	421–2203 ms	3.57	29.10
Incongruent	.92 (.87−.94)	.66 (.56−.75)	780 ms	425–2343 ms	2.78	17.60
TRCE	.57 (.36−.72)	.36 (.20−.50)	62 ms	– 236 to 683 ms	1.61	5.53
Error						
Congruent	.85 (.79−.89)	.66 (.54−.75)	4.3%	0–34%	2.27	9.71
Incongruent	.80 (.74−.84)	.44 (.28−.58)	14.9%	0–56%	.86	2.97
TRCE	.74 (.66−.80)	.46 (.30−.60)	10.7%	– 14 to 56%	.72	.09
Reactive						
Reaction time						
Congruent	.99 (.98−.99)	.67 (.43−.80)	1003 ms	501–2802 ms	2.54	12.40
Incongruent	.90 (.86−.94)	.60 (.40−.73)	1098 ms	510–3311 ms	2.22	10.40
TRCE	.59 (.42−.72)	.40 (.25−.54)	94 ms	– 642 to 967 ms	.78	3.97
Error						
Congruent	.86 (.78−.90)	.28 (.10−.44)	1.5%	0–31%	5.39	45.50
Incongruent	.86 (.80−.90)	.26 (.08−.43)	6.7%	0–56%	2.09	8.24
TRCE	.80 (.73−.86)	.18 (– .01 to .35)	5.1%	– 11 to 54%	2.16	6.25

*Note. N* = 128. *CI* confidence interval; TRCE = task-rule congruency effect. Split-half is an average of the test and retest phase split-half reliabilities. Test–retest = ICC (2,1)

**Table 8:** AX-continuous performance task baseline session reliability

Measure	Split-half (95% CI)	Test–retest (95% CI)	M	Range	Skew	Kurtosis
Reaction time						
AX trials	.98 (.97−.98)	.60 (.39−.74)	449 ms	295–827 ms	1.82	8.78
AY trials	.90 (.86−.93)	.72 (.61−.80)	540 ms	376–835 ms	1.74	8.60
BX trials	.89 (.86−.92)	.56 (.22−.74)	516 ms	267–1468 ms	1.45	5.45
BY trials	.98 (.98−.98)	.64 (.22−.82)	441 ms	273–788 ms	1.60	8.21
PBI	.72 (.62−.80)	.26 (.05−.44)	.03	– .40 to .24	– .29	– .27
BX interference	.71 (.60−.80)	.45 (.26−.60)	75 ms	– 109 to 872 ms	1.06	1.36
Error						
AX trials	.89 (.86−.92)	.15 (– .03 to .32)	6.6%	0–80%	1.03	3.31
AY trials	.45 (.27−.60)	.22 (.05−.38)	7%	0–44%	2.19	9.33
BX trials	.68 (.57−.76)	.30 (.07−.48)	13.8%	0–80%	1.12	3.77
BY trials	.64 (.48−.78)	.05 (– .12 to. 22)	1.1%	0–19%	.20	1.96
A no-go trials	.66 (.54−.74)	.20 (.03−.36)	11.1%	0–72%	.78	2.98
B no-go trials	.73 (.66−.80)	.28 (.09−.45)	22.3%	0–80%	4.25	28.00
PBI	.69 (.59−.76)	.15 (– .03 to .32)	– .18	– .94 to .89	.48	– 1.11
BX interference	.78 (.70−.84)	.39 (.17−.56)	2.85	– .23 to 4.4	.75	.13
*d′* context	.55 (.41−.66)	.23 (.05−.39)	.09	– 1.14 to .87	– .05	– .36
A-cue bias	.62 (.50−.72)	.27 (.06−.45)	1.08	– .52 to 2.83	– .08	– .62

*Note. N* = 112. *CI* confidence interval; *PBI* proactive behavioral index. Split-half is an average of the test and retest phase split-half reliabilities. Test–retest = ICC (2,1)

**Table 9:** AX-continuous performance task proactive session reliability

Measure	Split-half (95% CI)	Test–retest (95% CI)	M	Range	Skew	Kurtosis
Reaction time						
AX trials	.98 (.98−.99)	.80 (.73−.86)	415 ms	257–832 ms	1.45	6.27
AY trials	.88 (.82−.92)	.69 (.58−.78)	541 ms	378–871 ms	1.94	9.25
BX trials	.92 (.89−.94)	.68 (.56−.76)	460 ms	259–1010 ms	1.64	6.26
BY trials	.98 (.98−.99)	.79 (.70−.85)	410 ms	253–710 ms	1.43	7.46
PBI	.80 (.72−.85)	.51 (.36−.64)	.09	– .26 to .32	– .50	.64
BX interference	.77 (.69−.84)	.50 (.34−.62)	51 ms	– 91 to 493 ms	1.92	6.59
Error						
AX trials	.92 (.88−.94)	.54 (.40−.66)	5.7%	0–80%	.95	3.17
AY trials	.81 (.76−.86)	.27 (.08−.44)	18.6%	0–80%	1.87	7.17
BX trials	.67 (.56−.76)	.34 (.17−.49)	10.7%	0–56%	.75	2.63
BY trials	.58 (.41−.73)	.35 (.18−.49)	1.1%	0–15%	– .06	7.97
A no-go trials	.82 (.78−.88)	.38 (.20−.53)	17%	0–80%	1.24	3.93
B no-go trials	.82 (.77−.87)	.29 (.06−.48)	32%	0–80%	3.21	17.40
PBI	.80 (.73−.86)	.39 (.20−.56)	.16	– .89 to .94	– .25	– 1.20
BX interference	.80 (.72−.85)	.55 (.40−.66)	3.09	– .92 to 4.40	1.18	.90
*d′* context	.79 (.71−.85)	.42 (.24−.57)	.37	– 1.99 to 1.47	– .17	– .70
A-cue bias	.62 (.50−.73)	.29 (.11−.45)	.93	– .5 to 2.47	0.01	– .56

*Note. N* = 112. *CI* confidence interval; *PBI* proactive behavioral index. Split-half is an average of the test and retest phase split-half reliabilities. Test–retest = ICC (2,1)

**Table 10:** AX-continuous performance task reactive session reliability

Measure	Split-half (95% CI)	Test–retest (95% CI)	M	Range	Skew	Kurtosis
Reaction time						
AX trials	.98 (.98−.99)	.74 (.58−.83)	435 ms	259–923 ms	1.85	10.1
AY trials	.92 (.88−.94)	.69 (.51−.80)	558 ms	373–905 ms	1.31	6.13
BX trials	.89 (.86−.92)	.67 (.49−.78)	546 ms	336–993 ms	1.57	6.81
BY trials	.98 (.98−.99)	.76 (.55−.86)	420 ms	258–783 ms	1.36	7.26
PBI	.59 (.46−.70)	.43 (.27−.57)	.02	– .3 to .21	– .19	– .03
BX interference	.70 (.58−.78)	.50 (.35−.63)	125 ms	– 52 to 510 ms	.80	.94
Error						
AX trials	.84 (.78−.88)	.42 (.26−.55)	7.2%	0–47%	1.25	4.40
AY trials	.44 (.26−.59)	.28 (.10−.43)	7.0%	0–33%	1.87	7.50
BX trials	.75 (.66−.82)	.45 (.23−.61)	11.2%	0–78%	1.18	3.97
BY trials	.73 (.60−.82)	.19 (.01−.35)	1.2%	0–29%	.74	2.74
A no-go trials	.45 (.29−.59)	.44 (.28−.57)	8.4%	0–50%	1.03	3.19
B no-go trials	.59 (.46−.70)	.45 (.29−.58)	12.8%	0–56%	6.02	54.6
PBI	.65 (.54−.74)	.20 (.00−.38)	– .09	– .93. to .86	.22	– 1.50
BX interference	.79 (.72−.85)	.56 (.40−.68)	2.93	.58–4.4	1.04	.32
*d′* context	.53 (.39−.64)	.33 (.16−.48)	.06	– .8 to .82	– .12	– .54
A-cue bias	.72 (.62−.80)	.41 (.20−.57)	.93	– .27 to 3.18	– .28	– .35

*Note. N* = 112. *CI* confidence interval; *PBI* proactive behavioral index. Split-half is an average of the test and retest phase split-half reliabilities. Test–retest = ICC (2,1)

**Table 11:** Sternberg reliability across sessions

Measure	Split-half (95% CI)	Test–retest (95% CI)	M	Range	Skew	Kurtosis
Baseline						
NN rt	.95 (.91−.96)	.66 (.51−.77)	834 ms	466–1704 ms	1.79	7.13
NP rt	.94 (.92−.96)	.65 (.49−.76)	878 ms	444–1615 ms	1.24	4.97
RN rt	.78 (.70−.84)	.48 (.29−.64)	951 ms	492–1750 ms	1.11	4.06
Recency Eff rt	.08 (– .22 to .38)	.26 (.04−.45)	117 ms	– 201 to 480 ms	.54	.55
NN err	.71 (.57−.82)	.16 (– .07 to .37)	3.6%	0–56%	3.19	14.9
NP err	.76 (.67−.84)	.43 (.23−.60)	13.2%	0–58%	1.28	4.29
RN err	.05 (– .22 to .30)	.15 (– .05 to .35)	17.3%	0–60%	.77	3.14
Recency Eff err	– .21 (– .42 to .07)	.10 (– .10 to .30)	13.8%	– 12 to 60%	.77	.19
Proactive						
NN rt	.94 (.92−.96)	.66 (.51−.78)	834 ms	445–1477 ms	1.61	6.33
NP rt	.94 (.91−.96)	.66 (.48−.79)	845 ms	420–1505 ms	1.30	5.26
RN rt	.82 (.76−.88)	.65 (.42−.78)	1003 ms	448–1958 ms	.92	3.65
Recency Eff rt	.29 (.03−.51)	.34 (.14−.52)	169 ms	– 180 to 560 ms	.30	.61
NN err	.71 (.54−.81)	.14 (– .09 to .35)	5%	0–50%	2.10	9.64
NP err	.71 (.58−.81)	.25 (.03−.45)	12.4%	0–60%	1.20	4.39
RN err	.22 (– .04 to .45)	.15 (– .08 to .36)	25.6%	0–60%	.39	2.14
Recency Eff err	.01 (– .28 to .31)	.04 (– .19 to .26)	20.6%	– 25 to 60%	.31	– .78
Reactive						
NN rt	.87 (.81−.91)	.54 (.36−.68)	851 ms	460–1661 ms	1.61	6.08
NP rt	.94 (.92−.96)	.63 (.47−.75)	856 ms	482–1400 ms	1.26	5.09
RN rt	.91 (.88−.94)	.59 (.42−.72)	963 ms	491–1582 ms	1.11	4.20
Recency Eff rt	.19 (– .09 to .45)	.29 (.08−.48)	85 ms	– 176 to 350 ms	.24	1.05
NN err	.56 (.26−.74)	.17 (– .04 to .37)	4.3%	0–50%	2.52	9.39
NP err	.72 (.62−.81)	.35 (.15−.53)	10.3%	0–54%	1.48	5.55
RN err	.74 (.65−.82)	.48 (.29−.64)	12.7%	0–56%	1.01	3.54
Recency Eff err	.48 (.24−.66)	.24 (.03−.43)	8.3%	– 25 to 50%	N/A	N/A

*Note. N* = 104. *CI* confidence interval; *NN* novel negatives; *NP* novel positives; *RN* recent negatives. Split-half is an average of the test and retest phase split-half reliabilities. Test–retest = ICC (2,1)

## Appendix 2

Appendix Tables 12, 13 and 14

**Table 12:** Between-task Spearman rho correlations of selected measures, baseline session

Variable	*M*	*SD*	1	2	3	4	5	6	7	8	9	10	11
1. A-Cue	3.16	0.68											
2. BXI error	– 0.13	0.12	.19*										
3. BXI RT	67.47	71.71	.22*	– .00									
4. *d′*	2.84	0.76	.57**	.79**	.00								
5. PBI error	0.05	0.09	.16	– .86**	.14	– .63**							
6. PBI RT	0.04	0.07	– .25**	.18*	– .83**	.14	– .34**						
7. Recency error	0.13	0.10	– .01	– .25**	– .06	– .12	.21*	.05					
8. Recency RT	116.60	81.08	.13	– .04	– .00	.03	.08	– .10	.01				
9. Stroop error	0.03	0.04	– .27**	– .17	.01	– .27**	.07	– .03	– .01	.04			
10. Stroop RT	138.21	65.84	.09	– .18*	.10	– .12	.20*	– .09	– .02	.08	.10		
11. TRCE error	– 0.08	0.08	.24**	.18	.03	.24**	– .06	– .01	– .08	– .02	– .08	– .14	
12. TRCE RT	78.16	120.22	.15	.05	.12	.11	– .04	– .03	.01	.09	– .23*	.03	– .26**

*Note. N* = 120. M and SD are used to represent mean and standard deviation, respectively. *BXI* BX interference; *d′* d prime; *PBI* Proactive Behavioral Index; *Recency* recency effect; *TRCE* task-rule congruency effect. Test and retest phase combined

** *p* < .01; * *p* < .05

**Table 13:** Between-task Spearman rho correlations of selected measures, proactive session

Variable	*M*	*SD*	1	2	3	4	5	6	7	8	9	10	11
1. A-Cue	2.84	0.69											
2. BXI error	– 0.09	0.10	.21*										
3. BXI RT	48.35	64.98	.36**	– .22*									
4. *d′*	3.13	0.90	.53**	.82**	– .15								
5. PBI error	– 0.06	0.14	.34**	– .66**	.50**	– .54**							
6. PBI RT	0.09	0.09	– .34**	.37**	– .78**	.35**	– .72**						
7. Recency error	0.18	0.11	– .06	– .08	– .04	– .10	.06	– .07					
8. Recency RT	165.66	100.36	.02	.17	.11	.19*	– .20*	.02	– .00				
9. Stroop error	0.02	0.02	– .33**	– .15	– .02	– .33**	.02	– .02	.05	– .05			
10. Stroop RT	82.81	53.41	– .10	– .31**	.08	– .27**	.19*	– .17	– .11	.00	.29**		
11. TRCE error	– 0.13	0.10	.06	.11	– .04	.09	– .03	.03	– .11	.03	– .01	– .16	
12. TRCE RT	32.96	64.87	.05	– .15	.08	– .13	.18*	– .08	.08	– .03	– .12	.20*	– .37**

*Note. N* = 120. M and SD are used to represent mean and standard deviation, respectively. *BXI* BX interference; *d′* d prime; *PBI* Proactive Behavioral Index; *Recency* recency effect; *TRCE* task-rule congruency effect. Test and retest phase combined

** *p* < .01; * *p* < .05

**Table 14:** Between-task Spearman rho correlations of selected measures, reactive session

Variable	*M*	*SD*	1	2	3	4	5	6	7	8	9	10	11
1. A-Cue	3.08	0.66											
2. BXI error	– 0.10	0.12	.29**										
3. BXI RT	125.76	63.30	.22*	.07									
4. *d′*	2.94	0.85	.57**	.87**	.13								
5. PBI error	0.03	0.08	.02	– .85**	.01	– .76**							
6. PBI RT	0.02	0.05	– .15	.30**	– .64**	.25**	– .40**						
7. Recency error	0.08	0.09	– .05	– .24**	– .02	– .26**	.21*	– .11					
8. Recency RT	87.80	75.81	.10	.12	.15	.19*	– .10	– .04	– .10				
9. Stroop error	0.02	0.05	– .24**	– .32**	– .09	– .37**	.34**	– .06	– .07	.08			
10. Stroop RT	91.29	64.23	– .07	– .26**	.10	– .27**	.24**	– .19*	.13	.00	.44**		
11. TRCE error	– 0.05	0.05	.23*	.23*	.21*	.31**	– .19*	– .10	– .08	.03	– .16	– .17	
12. TRCE RT	59.67	132.11	– .02	– .03	– .02	– .03	.06	– .01	.03	– .06	– .06	.06	– .16

*Note. N* = 120. M and SD are used to represent mean and standard deviation, respectively. *BXI* BX interference; *d′* d prime; *PBI* Proactive Behavioral Index; *Recency* recency effect; *TRCE* task-rule congruency effect. Test and retest phase combined

** *p* < .01; * *p* < .05

## Appendix 3

### Overview of DMC task battery paradigms

#### Task paradigms

Here we present the most pertinent information regarding the tasks including their rationale. For a complete description of the tasks (e.g., ISI, etc.), see Tang et al. (2022).

***Stroop***

The color-word Stroop is widely recognized as a canonical task of cognitive control, in which top-down selective attention is required to focus processing on the task-relevant font color of printed words, while ignoring the irrelevant but otherwise dominant word name. A commonly used approach to manipulating cognitive control demands in the Stroop task is to vary *list-wide proportion congruence* (PC) (Lindsay & Jacoby, 1994; Logan & Zbrodoff, 1979). Under high list-wide PC conditions, congruent trials (word name matches font color, e.g., BLUE in blue font) are frequent and incongruent trials (word name indicates a different color than the font color, e.g., RED in blue font) are rare within a block, such that control demands are on average low and intermittent. In contrast, under low list-wide PC conditions (rare congruent trials, frequent incongruent), the high probability that interference will occur within a block should lead to an up-regulated cognitive control state.

In particular, we and others have hypothesized that under low list-wide proportion congruence (PC) conditions, the tendency to utilize *proactive control* will increase (Bugg, 2014; Bugg & Chanani, 2011). In this case, proactive control is theoretically associated with sustained maintenance of the task goal to attend to the ink color and ignore the word, which should be present in a consistent (i.e., global; present on all trials) and preparatory manner (i.e., engaged even prior to stimulus onset). Thus, the key prediction is that the Stroop effect (average slowing or increase in errors on incongruent relative to congruent trials) should be reduced on all trials, relative to a baseline, high list-wide PC condition, reflecting improved performance on incongruent trials and a reduction of facilitation on congruent trials (i.e., a congruency cost).

In contrast, proportion congruence (PC) can also be manipulated in an item-specific, rather than list-wide fashion (Jacoby et al., 2003). In this case, specific colors will occur with low PC (e.g., items appearing in green font will frequently be incongruent), while others may occur with high PC (e.g., items appearing in red font will frequently be congruent), and these “items” are randomly intermixed such that participants cannot predict whether a low PC or high PC item will appear on a given trial. This type of item-specific PC manipulation is theoretically predicted to enhance the utilization of *reactive control* for low PC items (Bugg & Dey, 2018; Bugg & Hutchison, 2013; Bugg et al., 2011). For these items, strong associations develop between a critical feature (a specific font color, such as green) and increased control demands (i.e., high interference), leading to more effective goal retrieval and utilization upon presentation of a stimulus that includes this feature (e.g., a word printed in a green font). The engagement of reactive control is expected to be transient, present only after stimulus onset, and only engaged by low PC incongruent items, particularly when these occur within the context of 50% congruent, or even higher, list-wide PC conditions.

The three Stroop task variants in the present battery varied as follows: the baseline condition had a high list-wide proportion congruence (PC) (67% congruent, 33% incongruent trials), whereas the proactive condition had a low list-wide PC (33% congruent, 67% incongruent trials). In contrast, the reactive condition approximated the high list-wide PC of the baseline condition (60% congruent, 40% incongruent) due to the inclusion of many high PC (100% congruent) filler items, but also featured specific items that were low PC (25% congruent, 75% incongruent). Another feature of the battery is the inclusion, in each condition, of *a set of unbiased, diagnostic items (“PC-50”, 50% congruent, 50% incongruent)* that did not share features (i.e., words or colors) with the other items in the condition*.* These PC-50 (diagnostic) items provide clearer behavioral markers from which to dissociate proactive and reactive control (Braem et al., 2019). Similar versions of these Stroop conditions have been examined in prior work, using both picture-word and color-word variants (Dey & Bugg, 2021). Finally, it is worth noting that because of the large numbers of different font colors (5) included in each of the conditions, the task was implemented with vocal rather than manual responding, using built-in voice recognition software to extract response latencies.

##### Baseline sessions

In a baseline session the trials were manipulated in a list-wide, mostly congruent (LW-MC) manner. Subjects completed a total of 288 trials during a baseline session, in which there were 96 PC-50 trials (48 congruent, 48 incongruent), and 192 biased trials. The biased set had 75% congruent (144 trials) and 25% incongruent (48 trials) trials. Consequently, the list-wide proportion congruency for the baseline sessions was 66%. The sessions were divided into two blocks of 144 trials each, between which subjects were instructed to rest for one minute.

##### Reactive sessions

In the reactive sessions the proportion congruency manipulation was at the item-level, item-specific proportion congruency (IS-PC). Thus for the biased set, purple and white color-font items were manipulated to be PC-25 (i.e., 25% congruent, 48 trials; 75% incongruent, 144 trials), while blue and red color-font items were manipulated to be PC-100 (i.e., these font-color words were only presented on congruent trials; 192 trials). Finally, as in the baseline and proactive sessions, the remaining 96 trials were PC-50 (i.e., equal amount of congruent and incongruent trials). Thus, subjects completed a total of 480 trials during the reactive sessions. Each reactive session was divided into three blocks of 160 trials each, between which subjects were instructed to rest for one minute.

##### Proactive sessions

In the proactive sessions, the trials were manipulated in a list-wide, mostly incongruent (LW-MI) manner. Subjects completed a total of 288 trials during the proactive sessions, in which there were 96 trials PC-50 (48 congruent, 48 incongruent), and 192 biased trials. The biased set had 25% congruent (48 trials) and 75% incongruent (144 trials) trials. Consequently, the list-wide proportion congruency for the proactive sessions were 33%. A proactive session was divided into two blocks of 144 trials each, between which subjects were instructed to rest for one minute.

##### Cognitive control measures

Average reaction times (RTs) on correct trials and error rates were calculated for both congruent and incongruent trials for the biased set, for each subject in each session. The Stroop interference effect (incongruent–congruent) in both RT and also error rate was calculated separately for biased items. For brevity, the results of the PC-50 item set are not reported.

***AX-CPT***

The AX-CPT has become increasingly utilized as a task of context processing and cognitive control, given its simplicity, flexibility and applicability in a wide-range of populations (Barch et al., 2008; Chatham et al., 2009; Chun et al., 2018; Janowich & Cavanagh, 2018; Servan-Schreiber et al., 1996). In the paradigm, participants respond to letters presented one at a time, with each trial consisting of a cue-probe letter pair. When an A-cue is followed by an X-probe, a target response is required. Since the AX pairing occurs frequently, strong cue-probe associations develop. Cognitive control is postulated to be needed to maintain and utilize the information provided by contextual cues, particularly to minimize errors and response interference occurring on BX trials (where B refers to any letter except A), which occur when the X-probe is presented, but is not preceded by an A-cue. In prior work, shifts in the tendency to utilize *proactive* or *reactive control* have not only been observed when comparing different populations or groups, but have also been manipulated within-subjects (Braver et al., 2009).

The AX-CPT conditions included in the battery extend prior recent work using a task variant in which the A- and B-type contextual cues occur with equal frequency, thus eliminating confounds in earlier versions that could be due to the lower overall frequency of encountering B-cues (Gonthier et al., 2016; Richmond et al., 2015). Further, these conditions also include no-go trials, in which the probe is a digit rather than letter. Because of the increase in response uncertainty (i.e., three types of probe response are possible: target, nontarget, no-go), the addition of no-go trials decreases the overall predictive utility of context information for responding, and as a consequence was found to reduce the overall proactive control bias typically observed in healthy young adults. As such the no-go conditions result in a “low control” baseline, from which to more sensitively observe condition-related changes in control mode (Gonthier et al., 2016). In all of the current AX-CPT versions tested in this battery, the task structure, trial types and frequencies are identical, except for the specific manipulations described below for proactive and reactive conditions.

The *proactive* condition replicates prior work using context strategy training (Gonthier et al., 2016), as a means of increasing the predictive preparation of responses following contextual cue information. Specifically, participants are provided with explicit information regarding the frequencies of these cue-response associations, and receive training and practice in utilizing them to prepare the dominant responses. In addition, during inter-trial intervals, participants are provided with visual instructions to “remember to use the strategy”. The key prediction is that the increased utilization of contextual cue information will lead to a bias to prepare a target response following an A-cue (analyzed in terms of both AX and AY trials) and a nontarget response following a B-cue, leading to reduced interference on BX trials. Yet a side effect of this preparatory bias is a predicted increase in errors and response interference on AY trials, which occur when the A-cue is *not* followed by an X-probe.

The *reactive* condition involved a new manipulation which has not previously been examined in prior work. Specifically, the reactive condition utilizes context-specific probe cueing (similar to other context cueing manipulations in tasks, such as Stroop and flanker; for review, see (Bugg & Crump, 2012)), in that for high control demand trials (AY, BX, no-go) the probe item appears in a distinct spatial location, and with a distinct border color surrounding it (presented briefly before the onset of the probe). Critically, because these featural associations are only present at the time of probe onset, they were not hypothesized to modulate the utilization of proactive control strategies. Likewise, the probe features could not drive direct stimulus-response learning, since they do not directly indicate the appropriate response to be made. In other words, the probe feature cannot be used as a “stop signal”, since on high control demand trials it signals the need for a go response as often as a no-go. Likewise, on low control demand trials, the probe feature predicts a target response (when it follows an A-cue) as often as it does a non-target response (when it follows a B-cue). In contrast, the probe features do serve as contextual cues signaling high control demand, and thus prompt more rapid and effective retrieval of contextual information to resolve the conflict. Because information about high-conflict probe features is not provided explicitly to participants (in contrast to the proactive condition), it has to be learned implicitly through experience. The key prediction is that utilization of probe features should reduce the tendency to make BX errors but could increase BX reaction time interference (due to the tendency to utilize the probe to drive context retrieval).

##### Baseline sessions

For all AX-CPT sessions, the task comprised 216 trials total, and included 72 AX trials, 72 BY trials, 18 AY trials, 18 BX trials and 36 no-go trials (18 following an A-cue, 18 following a B-cue). All trial types and no-go trials were presented in random order. The task was performed in three 72 trial blocks, between which subjects were instructed to take a minimum of 1-minute rest break. After receiving task instructions, subjects performed a 12-trial practice block before beginning the actual task.

##### Reactive sessions

The occurrence of high conflict trials (AY, BX, no-go) was implicitly signaled by presenting the probe in a distinct spatial location and preceded by a distinct border color. Specifically, while cues were always presented centrally (as in the baseline and proactive variants) the probe stimuli were either presented in the upper half (AX, BY) or lower half (AY, BX, no-go) of the visual display. Furthermore, probe stimuli were immediately preceded (250 msec before probe onset) by either a white border (AX, BY) or red border (AY, BX, no-go). Otherwise, the task structure and trial proportions were identical to baseline and proactive variants.

##### Proactive sessions

In the proactive sessions, subjects received strategy training before completing the AX-CPT. The strategy training occurred during a practice block of six trials, during which an audio clip was played, which instructed subjects which button to prepare following the cue. After this first series of practice trials, subjects performed a second practice set (six trials), during which they were asked to type which button they were preparing to press in response to the second item. Subjects typed out “left” or “right” and the program told subjects if they were correct or not. If they were not correct, they were reminded what letter the first item was and asked to try again. This procedure was implemented to accommodate the online testing format, and deviated slightly from in-person versions, in which subjects responded verbally regarding the button they were preparing to press.

##### Cognitive control measures

Average reaction times (RTs) on correct trials and error rates were calculated for each of the four primary trial types (AX, AY, BX, BY) for each subject in each session. Average error rates for no-go trials were calculated as well. Additional derived indices were also computed: A-cue bias, *d’*-context, the Proactive Behavioral Index (PBI), and BX probe Interference (Gonthier et al., 2016). The first two indices, A-cue bias, and d’-context are based on signal detection theory, (Stanislaw & Todorov, 1999) and reflect the use of proactive control. The A-cue bias measure was calculated by computing a *c* criterion from hits on AX trials and false alarms on AY trials as 1/2*(Z[H] + Z[F]), with H representing hits on AX trials and F representing false alarms on AY trials (Richmond et al., 2015). The *d’*-context index was calculated by computing a *d’* index from hits on AX trials and false alarms on BX trials as Z(H) – Z(F), with H representing hits on AX trials, F representing false alarms on BX trials, and Z representing the z-transform of a value. The third index was the PBI, calculated as (AY – BX)/(AY + BX) (Braver et al., 2009). This index reflects the relative balance of interference between AY and BX trials; a positive PBI reflects higher interference on AY trials, indicating proactive control, whereas a negative PBI reflects higher interference on BX trials, indicating reactive control. The PBI was computed separately for error rates (based on average error rates on AY and BX trials) and for RTs (based on average RTs on AY and BX trials). The fourth index was BX probe interference, calculated as (BX – BY) on both error rates and RTs, including a standardized RT computation. This index allows for examination of the interference that occurs when an “X” probe follows a non-target cue “A”, and a target trial response must be inhibited.

To correct for error rates that were equal to 0, a log-linear correction was applied to all error rate data prior to computing the *d’*-context, the A-cue bias, PBI, and BX interference (Braver et al., 2009; Hautus, 1995). Although commonly such correction is only applied on indices stemming from signal detection theory, it is technically possible to produce calculation errors in PBI (i.e., AY – BX / AY + BX) due to dividing by 0; some subjects achieved an error rate of 0 on both AY and BX trials. In the calculation of BX interference (i.e., BX – BY), no such calculation error can occur. However, due to our interest in the correlations between these measures, we decided to apply the correction on BX interference as well. The correction was applied as

$$error+0.5/ Number\ of\ observations+1$$

***Cued task-switching***

Cued task-switching (Cued-TS) has long been recognized as a critical paradigm to assess a core component of cognitive control – the ability to activate and update task-representations in an on-line manner, in order to configure attention and action systems to process the task-relevant features of a current target. The key aspect of the paradigm is that two or more tasks randomly alternate across trials, with target items typically being ambiguous, so that they can be processed according to multiple task rules. Consequently, the advance presentation of the task cue, prior to target onset, is what disambiguates the target and specifies the appropriate stimulus-response rules.

An important metric of cognitive control in task-switching paradigms is the task-rule congruency effect (TRCE), which refers to the increased interference (both errors and reaction time) when the target response required for the current task is incongruent with the response that would be required to the same target stimulus if the alternative task had been cued (Meiran & Kessler, 2008). Consider the letter-digit task-switching (also called consonant-vowel, odd-even [CVOE]) task comprising a letter task and a digit task. If in the letter task, a right button press is required for a consonant and a left button press for a vowel, while in the digit task, a right button press is required for odd and a left button press for even, the “D4” target stimulus would be incongruent (whereas the “A2” target stimulus would be congruent, since for either task, the left button press would be correct). There is an extensive literature on the TRCE beginning with Sudevan and Taylor (1987), which includes work showing that this metric is quite sensitive to prefrontal cortex lesions (Aron et al., 2004) and activation (Konishi et al., 2003), and shows provocative differences between human and non-human primates (Stoet & Snyder, 2003) indicating its utility as a measure of cognitive control. Two additional important metrics are switch costs, which refer to the decrement to performance when the task to be performed on the current trial switches from that on the previous trial (relative to task-repeats, when the same task is performed on two consecutive trials) (Meiran, 1996; Rogers & Monsell, 1995), and mixing costs, which refer to the decrement to performance that occurs on task-repeat trials (relative to performance within a single-task block) (Braver et al., 2003; Los, 1996). These have also served as indices of cognitive control demands.

In prior work, including reward incentives on a subset of trials, with reward cues presented at the time of the task cue, led to a strong reduction in the mixing cost – and this was present even on the trials that were non-incentivized – but there was no effect on the task-rule congruency effect (TRCE) (Bugg & Braver, 2016). This finding was interpreted as indicating that the mixing cost reductions reflected a list-wide (global) enhancement of *proactive control*, whereas the TRCE effect is primarily influenced by *reactive control*, and so less impacted by advance reward incentive manipulations. The Cued-TS conditions included in the current battery build on this prior work by using variants of the consonant-vowel, odd-even (CVOE) (letter/digit) paradigm that aim to accentuate the robustness of the TRCE, while also enabling clear utilization of *proactive control* through the use of advance task cues with a long cue-to-target interval (CTI). A robust finding from prior work is that performance improves with longer preparation times (CTI), suggesting advanced preparation for relevant task rules and stimulus-response mappings for the upcoming target (Meiran, 1996).

In the baseline condition, target stimuli are list-wide mostly congruent (67%), as prior work has found that mostly congruent conditions result in a large and robust task-rule congruency effect (TRCE) (Bugg & Braver, 2016). The *proactive* condition builds on Bugg and Braver (2016) in keeping the same list-wide mostly congruent structure as the baseline condition but adding reward incentives on a subset of trials. Specifically, on 33% of trials, reward cues are presented simultaneously with advance task cues (i.e., by presenting the task cue in green font), and indicate the opportunity to earn monetary bonuses if performance is accurate and fast (relative to baseline performance) on that trial. By only presenting reward cues on a subset of trials, the remaining subset of non-incentivized trials and target stimuli can be directly compared across the proactive and baseline conditions. A divergence from Bugg and Braver (2016) is that single-task conditions are not included as part of the battery (due to length constraints), which precludes direct calculation of mixing costs. Nevertheless, the key prediction is that enhanced *proactive control* will lead to a global improvement of performance (i.e., faster RTs without a loss in accuracy).

The *reactive* condition utilizes a new manipulation which has not previously been examined in prior work. Specifically, the reactive condition includes punishment (rather than reward) incentives, again on the same 33% subset of trials that were incentivized in the proactive condition. However, in the reactive condition the incentive cue is presented at the time of the target stimulus, rather than with the task cue, which precludes the use of incentive motivation in a preparatory fashion. Participants are instructed that they will lose a component of their potential monetary bonus if they make an error on these incentivized trials. Critically, the incentivized trials occur preferentially (75%) with incongruent target stimuli. This manipulation is intended to associate punishment-related motivation with these high-conflict items, potentially leading to increased response monitoring and caution when incongruence is detected. As such, the key prediction is that enhanced reactive control should reduce the error task-rule congruency effect (TRCE), even on the non-incentivized trials, when compared to baseline and proactive conditions. Conversely, the RT TRCE should be increased, due to the tendency to utilize target features (detection of incongruency) to drive retrieval of task rules.

The target stimuli were constructed in terms of two distinct stimulus sets. One set of stimuli (A1, A2, B1, B2, 1A, 2A, 1B, 2B) were kept mostly congruent (80% congruent; 20% incongruent), also referred to as the biased set. The second set of stimuli (D4, E3, H5, I6, 4D, 3E, 5H, I6) were unbiased (50% congruent, 50% incongruent). Each session consisted of 192 total trials, 96 mostly congruent (80 congruent, 16 incongruent) and 96 unbiased (48 congruent, 48 incongruent) and also equally split between the two tasks (i.e., 96 letter, 96 digit). Trials were separated into three 64 trial blocks, between which subjects were required to take a minimum of 1-min rest break. Prior to starting each session subjects learned (or refreshed their memory) of the task rules through a set of 16 practice trials.

##### Baseline sessions

For the baseline session, no manipulations were made to the unbiased stimuli. However, to maintain consistency with the proactive and reactive sessions described below, for these stimuli task cues and target stimuli could appear in either red or green font. However, this distinction was irrelevant with regard to the instructions given to the subjects.

##### Reactive sessions

The reactive sessions of Cued-TS were identical to the baseline variant except for the addition of a punishment-based motivational incentive. This motivational incentive provides subjects with a punishment cue indicated during presentation of the target. When subjects made errors on incentive trials, which were indicated by a green cue and target, they received a monetary penalty for that trial that was subtracted from their compensation amount.

##### Proactive sessions

The proactive sessions of Cued-TS were identical to the baseline sessions except for the addition of a reward-based motivational incentive. This motivational incentive provides subjects with a reward cue, indicated by a cue in green font-color during presentation of the task cue. Non-incentive trials indicated by the task cue appearing in red font. When subjects responded to incentive trials faster than the baseline session’s median RT while maintaining accuracy (this information was stored in a look-up table database, and accessed at the beginning of each session), they received a monetary bonus for that trial added to their compensation amount.

##### Cognitive control measures

Average reaction times (RTs) on correct trials and error rates were calculated separately for congruent/incongruent biased items, for each subject in each session. Additionally, the TRCE (task-rule congruency effect) was calculated as a difference score between incongruent and congruent trials and was computed for biased items. A congruency effect was chosen over switch cost for two reasons: one, the task-rule congruency effect was closer in essence to the other effects (i.e., Stroop effect, interference effect, and recency effect), all of which are calculated as the difference score between an incongruent and a congruent condition, and two, in a preliminary round of calculations, the traditional reliability of the switch costs was much worse than the reliability for the congruency effect. For brevity, the results of the unbiased set are not reported.

***Sternberg***

The Sternberg item-recognition task has been one of the most popular experimental paradigms used to assess short-term / working memory for over 50 years (Sternberg, 1966), but more recently has been adapted particularly for the study of cognitive control with the “recent probes” version (Jonides & Nee, 2006). Like standard versions of the paradigm, the recent probes version presents participants with a memory set of various load levels (number of items), to maintain over a short delay (retention period), after which a single item probe is presented, which requires a target response if the probe was a part of the memory set. A classic finding in the literature is that as the memory set increases in size, WM load increases, and performance declines accordingly (higher error rates, longer RTs) (Shiffrin & Schneider, 1977; Sternberg, 1966). Under conditions in which the WM load is below capacity (3–4 items), active maintenance and rehearsal processes can be used to keep the memory set accessible, as an attentional template from which to prospectively match against the probe item (i.e., utilizing *proactive control* strategies). In contrast, when the WM load is above capacity (~ 7 items), probe responses are likely to be driven by retrieval-focused processes, such as familiarity (i.e., *reactive control* strategies).

In recent probes versions, the key manipulation is that the probe item can also be a part of the memory set of the previous trial, but not the current trial, which is termed a “recent negative” (RN) probe. On these RN trials, the probe is associated with high familiarity, which can increase response interference and errors, unless cognitive control is utilized to successfully determine that the probe familiarity is a misleading cue regarding its status (target or nontarget). The current versions of the Sternberg WM paradigm included in the battery are adapted from previous studies (Burgess & Braver, 2010; Speer et al., 2003), in using manipulations of WM load expectancy and RN frequency. Specifically, in all conditions, trials randomly vary in set size, with words used as stimuli, such that all items are novel on each trial, with the exception of RN probes. Under such conditions, Burgess & Braver (2010) found strong RN interference effects in both RT and errors. Likewise, following Speer et al. (2003), the set size in a given trial is revealed sequentially, leading to unpredictability and reliance on WM load expectancies to engage control strategies.

In the baseline condition, most trials have high WM load (6–8 items; 60%) and recent negative (RN) frequency is low (20% of nontarget probes), which should reduce tendencies to engage either proactive or reactive control strategies. However, in the *proactive* condition, most trials have low WM load (2–4 items; 60%), leading to the expectancy that active maintenance-focused and proactive attentional strategies will be effective, while RN frequency remains low (matched at 20% nontarget probes), such that the utility of reactive control should be unchanged. The critical prediction concerns the five-item set size, which occurs equivalently in all conditions (40% of trials), and thus can be equivalently compared between them. The key hypothesis is that use of proactive control strategies will improve both RT and accuracy, primarily for the target probe items (termed novel positive, or NP, since they never overlap across trials).

In the *reactive* condition, WM loads are identical to the baseline condition, while the frequency of recent negative (RN) trials is increased (80% of nontarget probes). Thus, in the reactive condition, it is familiarity-based interference expectancy that increases, rather than WM load expectancy. Based on the increased interference-expectancy, the theoretical hypothesis is that participants will not rely on familiarity as a cue for responding, and will rather evaluate the match of the probe to items stored in WM. Consequently, the key prediction is that performance on RN (or rather the RN effect, computed by subtracting performance on novel negative or NN trials) will be significantly improved relative to baseline.

##### Baseline sessions

The baseline sessions involved high-load variable-items and a low proportion of recent negative (RN) trials (20% of negative probes, 10% of total trials). Specifically, the variable-load set consisted of a mixture of high-load memory sets (12 six-item, 24 seven-item, 36 eight-item) and very few RN trials (four RN, 32 novel negative (NN), 36 novel positive (NP)). For the critical five-item set, the proportion was slightly adjusted, to increase the number of RN trials for analysis (eight RN, 16 NN, 24 NP).

##### Reactive sessions

In the reactive sessions, the variable-load set used the identical mixture of high-load memory set items as the baseline session (12 six-item, 24 seven-item, 36 eight-item). However, the relative proportion of RN to NN trials was increased in both the variable-load (32 RN, four NN, 36 NP) and critical items (16 RN, eight NN, 24 NP).

##### Proactive sessions

In the proactive sessions, the variable-load items were instead a mixture of low-load memory sets (36 two-item, 24 three-item, 12 four-item). The proportion of RN, NN, and NP trials was identical to the baseline session for both variable-load (four RN, 32 NN, 36 NP) and critical item sets (eight RN, 16 NN, 24 NP).

##### Cognitive control measures

Average reaction times (RTs) on correct trials and error rates were calculated per trial type (i.e., NN, NP, RN trials) for critical items (list-length 5). One additional index, the recency effect, was also calculated for both RTs and error rates as a difference score on negative trials as RN trials – NN trials. For brevity, the results of the variable-load item set are not reported here.

## Appendix 4

### Effect of age on analyses

#### Test–retest reliability and age

Our results indicate a stark increase in test–retest reliability when switching from traditional methods (MPE) to hierarchical Bayesian methods (HBM). Additional analyses were conducted to test whether these results were biased by the effects of age-related cognitive slowing. Our test–retest reliability analyses show an age-insensitivity when comparing a group consisting of all subjects (*N* ~ 110, aged 22–64) to a subset of that group (*N* ~ 80, aged 22–40) of subject 40 years old and younger (see Fig. Appendix Fig. 6). Besides the baseline sessions across the AX-CPT and Task Switching tasks, the measures show no difference between the two age groups. Moreover, the primary finding showing a substantial increase in test–retest reliability from the HBM approach, relative to MPE, is still present for all measures, even when focusing on a younger adult sample.

## Footnotes

1 We examined the effect of age on our analyses and have included them in Appendix 4

2 Control corresponds to non-interference trial types (e.g., Stroop congruent, Sternberg novel negative). Interference corresponds to interference trial types (e.g., AX-CPT AY and BX, task-switching incongruent).

3 Based on a reviewer suggestion, we examined the possibility that these results might have been biased by including data from older adults, as a consequence of age-related slowing. We conducted the same HBM test–retest reliability analyses after excluding individuals over 40 and found a very similar pattern of results. These are reported in Appendix 4.

## Code Availability

The analysis scripts (R) that pre-processed the raw data (available; see above) and generated the results and figures of the current study are available in the Snijder et al., 2022 repository, https://osf.io/pqvga/.
